# Spectroscopic Study on the Interaction between Naphthalimide-Polyamine Conjugates and Bovine Serum Albumin (BSA)

**DOI:** 10.3390/molecules200916491

**Published:** 2015-09-11

**Authors:** Zhi-Yong Tian, Li-Na Song, Yuan Zhao, Feng-Lei Zang, Zhong-Hua Zhao, Nan-Hao Chen, Xue-Jun Xu, Chao-Jie Wang

**Affiliations:** 1 Institute of Chemical Biology, Henan University, Kaifeng 475004, China; E-Mails: tzynew@163.com (Z.-Y.T.); 13674974223@163.com (L.-N.S.); Zangfenglei@sohu.com (F.-L.Z.); Zhaozhonghua@sohu.com (Z.-H.Z.); 2 The Key Laboratory of Natural Medicine and Immuno-Engineering, Henan University, Kaifeng 475004, China; E-Mail: zhaoyuanchem@study.xmu.edu.cn; 3 School of Pharmaceutical Sciences, Sun Yat-Sen University, Guangzhou 510006, China; E-Mail: beyondkaisas@gamail.com.; 4 Huaihe Clinical Institute, Henan University, Kaifeng 475004, China

**Keywords:** naphthalimide-polyamine, conjugates, bovine serum albumin (BSA), spectroscopic methods, molecular docking

## Abstract

The effect of a naphthalimide pharmacophore coupled with diverse substituents on the interaction between naphthalimide-polyamine conjugates **1**–**4** and bovine serum albumin (BSA) was studied by UV absorption, fluorescence and circular dichroism (CD) spectroscopy under physiological conditions (pH = 7.4). The observed spectral quenching of BSA by the compounds indicated that they could bind to BSA. Furthermore, caloric fluorescent tests revealed that the quenching mechanisms of compounds **1**–**3** were basically static type, but that of compound **4** was closer to a classical type. The *K_sv_* values at room temperature for compound-BSA complexes-**1**-BSA, **2**-BSA, **3**-BSA and **4**-BSA were 1.438 × 10^4^, 3.190 × 10^4^, 5.700 × 10^4^ and 4.745 × 10^5^, respectively, compared with the value of MINS, 2.863 × 10^4^ at Ex = 280 nm. The obtained quenching constant, binding constant and thermodynamic parameter suggested that the binding between compounds **1**–**4** with BSA protein, significantly affected by the substituted groups on the naphthalene backbone, was formed by hydrogen bonds, and other principle forces mainly consisting of charged and hydrophobic interactions. Based on results from the analysis of synchronous three-dimensional fluorescence and CD spectra, we can conclude that the interaction between compounds **1**–**4** and BSA protein has little impact on the BSA conformation. Calculated results obtained from *in silico* molecular simulation showed that compound **1** did not prefer either enzymatic drug sites I or II over the other. However, DSII in BSA was more beneficial than DSI for the binding between compounds **2**–**4** and BSA protein. The binding between compounds **1**–**3** and BSA was hydrophobic in nature, compared with the electrostatic interaction between compound **4** and BSA.

## 1. Introduction

**Figure 1:**
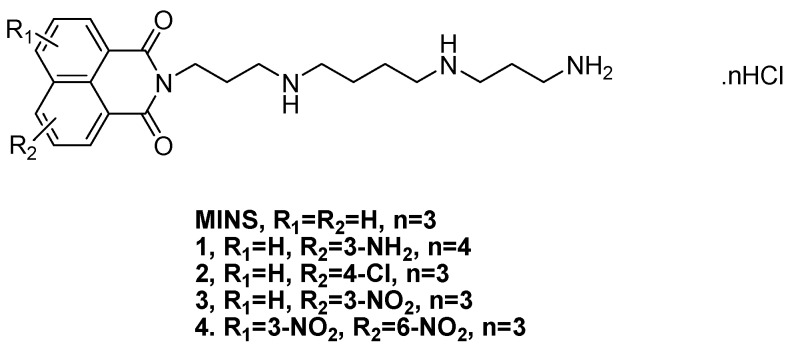
Structures of naphthalimide-polyamine conjugates.

Proteins are important chemical substances in cellular life and a major target for many types of medicine; furthermore, changes of their contents in serum can display human health conditions [1,2]. Serum albumin (SA), including bovine serum albumin (BSA) and human serum albumin (HSA), is the main soluble protein constituent of the circulatory system. SA plays an important and efficient role in drug delivery because of its ability to reversibly bind to a large variety of exogenous compounds, including fatty acids, amino acids, drugs and pharmaceuticals [3,4,5]. Due to its medical importance, low cost, ready availability, unusual ligand-binding properties and similarity to human serum albumin (HSA) in terms of spatial structure and chemical composition [6,7,8], BSA was selected as the model protein for this research. It is well known that 1,8-naphthalimide derivatives can intercalate DNA base pairs and bind to SA [2,9,10]. Similarly, polyamines can also bind to SA [11,12,13,14]. Naphthalimide-polyamine conjugates have been proven to exhibit good activity *in vitro* [15,16,17,18], intercalate DNA base pairs, and cause conformational variations in DNA [19]. MINS, a mononaphthalimide-polyamine conjugate, can bind to BSA, but causes a weak conformational change in BSA [20]. In this study, the impact of naphthalimide coupled with diverse substituent groups on the interaction between naphthalimide-polyamine conjugates **1**–**4** (Figure 1) and BSA was examined through ultraviolet (UV), fluorescence (FL), and circular dichroism (CD) spectroscopy and molecular modeling methods. The binding constants and main types of binding force therein were also investigated.

## 2. Results and Discussion

### 2.1. UV Spectroscopic Characteristics

**Figure 2:**
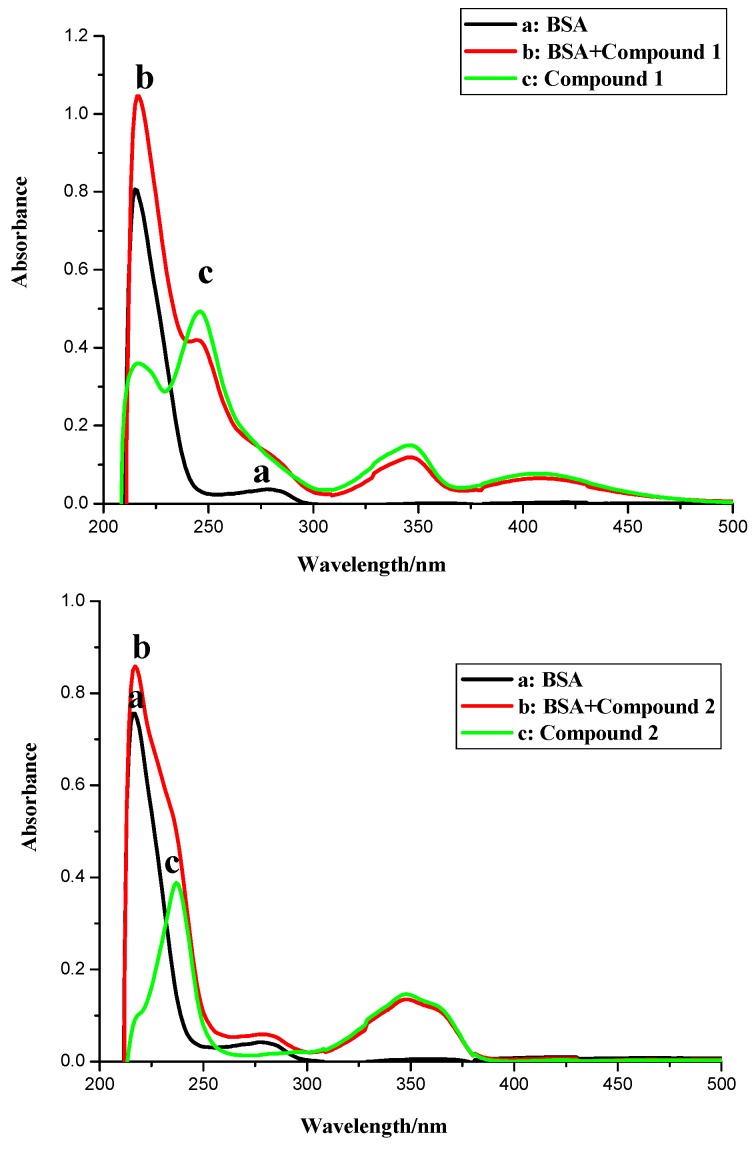

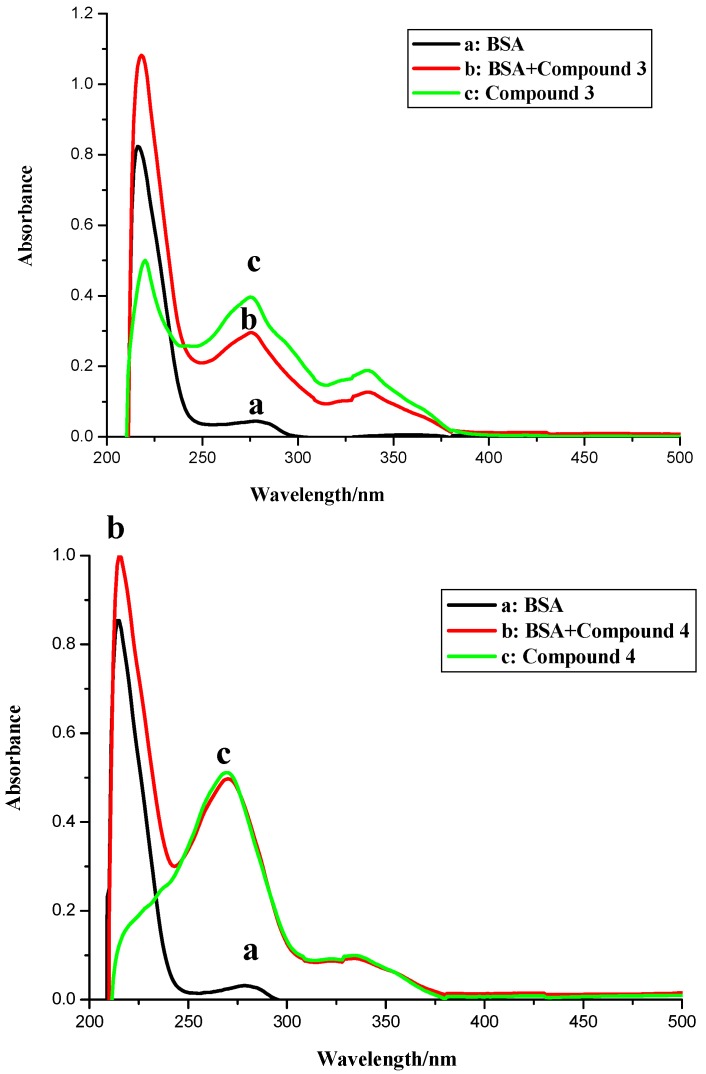
UV of compounds **1**–**4**, BSA and the BSA-compound complexes. Conditions: *c* (compound) = 12 × 10^−6^ mol∙L^−1^; *c* (BSA) = 1.04 × 10^−6^ mol∙L^−1^.

**Figure 3:**
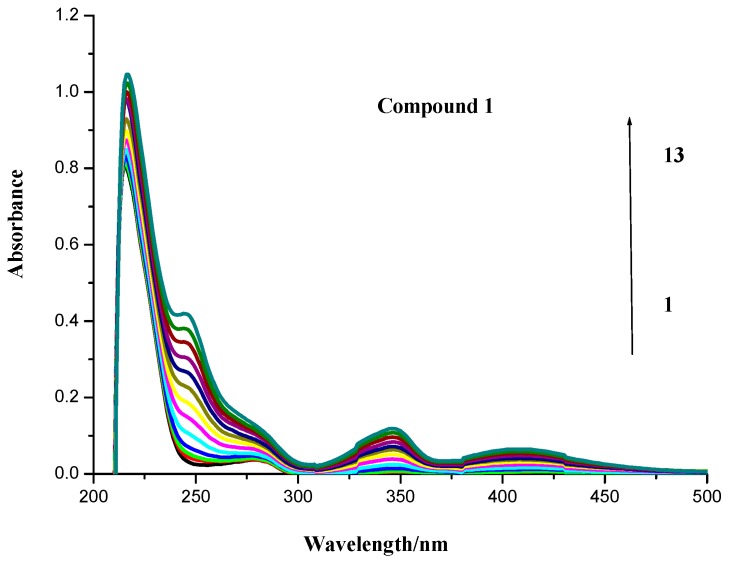

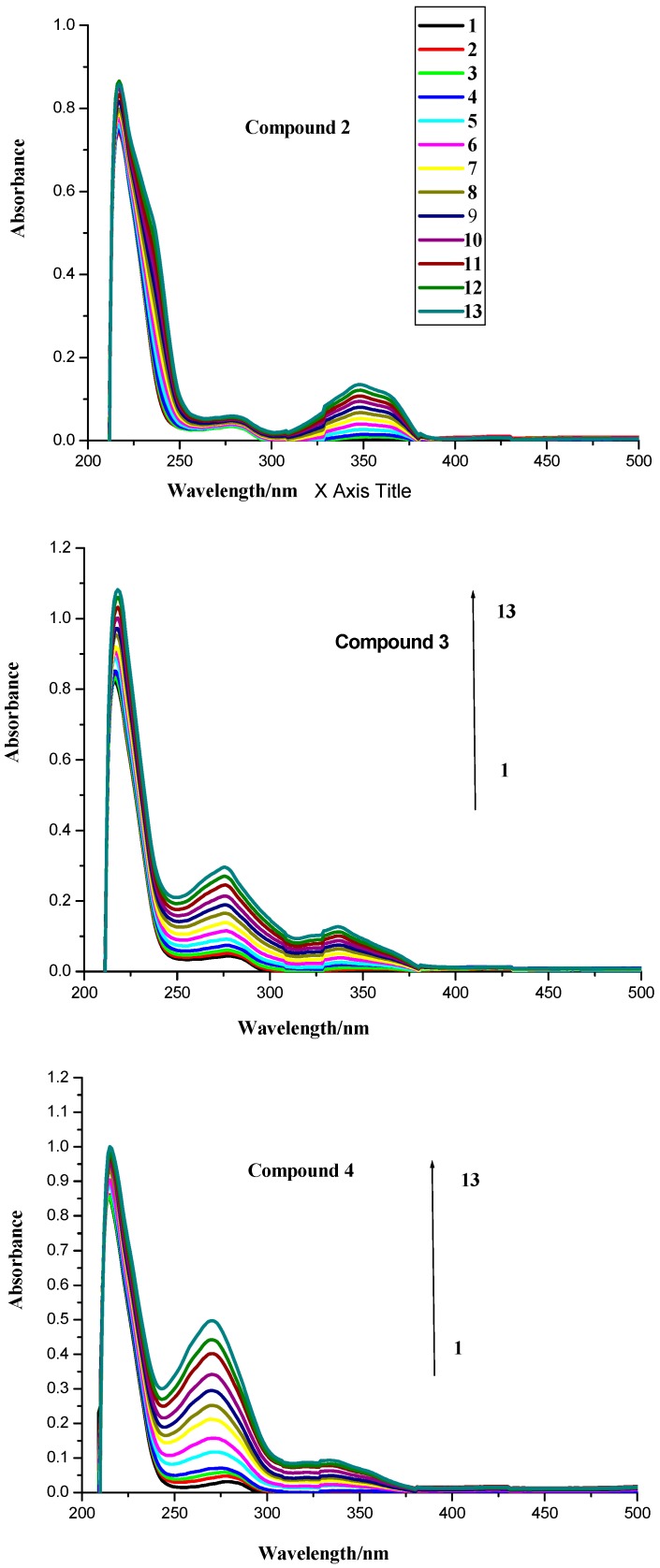
UV absorption spectra of compounds **1**–**4** with BSA. Numbers 1–13 indicated the concentrations of compounds **1**–**4**: 0.0, 0.4 × 10^−6^, 0.8 × 10^−6^, 1.2 × 10^−6^, 2.4 × 10^−6^, 3.6 × 10^−6^, 4.8 × 10^−6^, 6.0 × 10^−6^, 7.2 × 10^−6^, 8.4 × 10^−6^, 9.6 × 10^−6^, 10.8 × 10^−6^ and 12 × 10^−6^ mol∙L^−1^, respectively. BSA concentration applied was 1.04 × 10^−6^ mol∙L^−1^.

As shown in Figure 2, the UV spectra of BSA in the presence of different concentrations of compounds **1**–**4** were measured by an ultraviolet-visible range spectrophotometer. The free BSA had a peak at 278 nm (Figure 2), which was obviously increased with elevated concentrations of compounds **1**, **3** and **4** (Figure 3). However, it could not be deduced directly that the BSA complexes with compounds **1**, **3** and **4** were formed due to the overlapped absorptions of these compounds and BSA in the same region (Figure 2). Compound **2** had no peak at 278 nm. Although the addition of compound **2** to BSA led to a continuous increase to a lesser extent (Figure 3), the compound-BSA complex might have been formed because of the up-regulated absorption at 278 nm (Figure 3). At least, the spectral result of compound **2** indicated that it penetrated the hydrophobic sites of BSA sub-domain and bonded with the chromophores of tyrosine and tryptophan residues, and other unbonded residues were buried in a hydrophobic cave [21]. Interestingly, the gains in the absorption intensity of the postulated complexes of compounds **1**, **3**, **4** with BSA at 278 nm were less than expected (Figure 2). More evidence is needed to confirm the binding of these three compounds to BSA.

### 2.2. Fluorescence Spectroscopy

#### 2.2.1. Fluorescence Quenching

**Figure 4:**
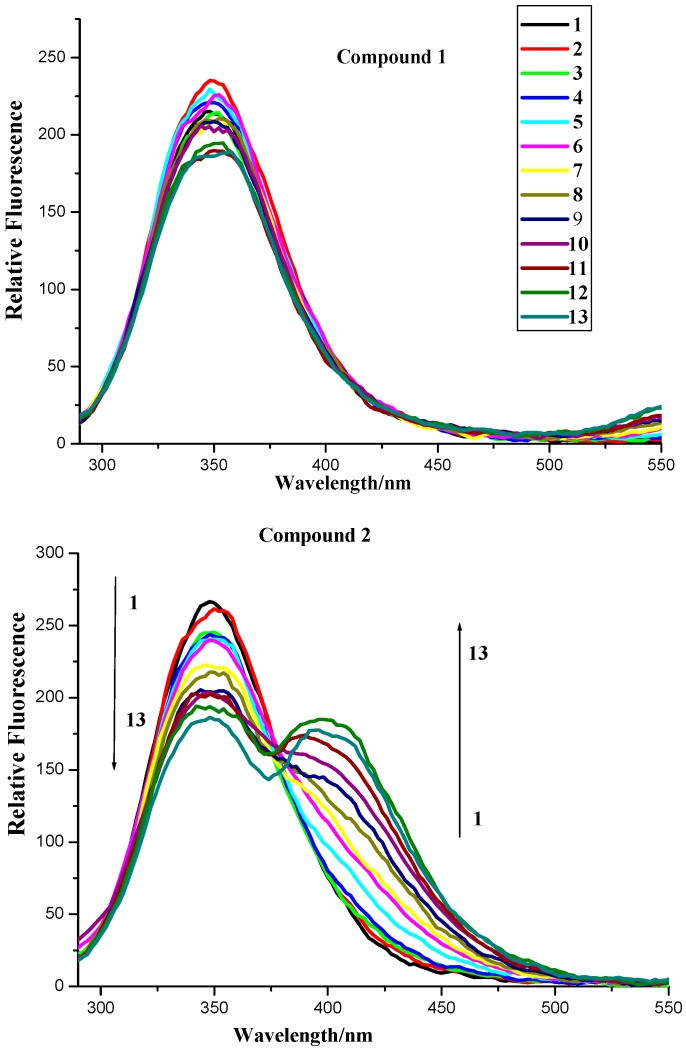

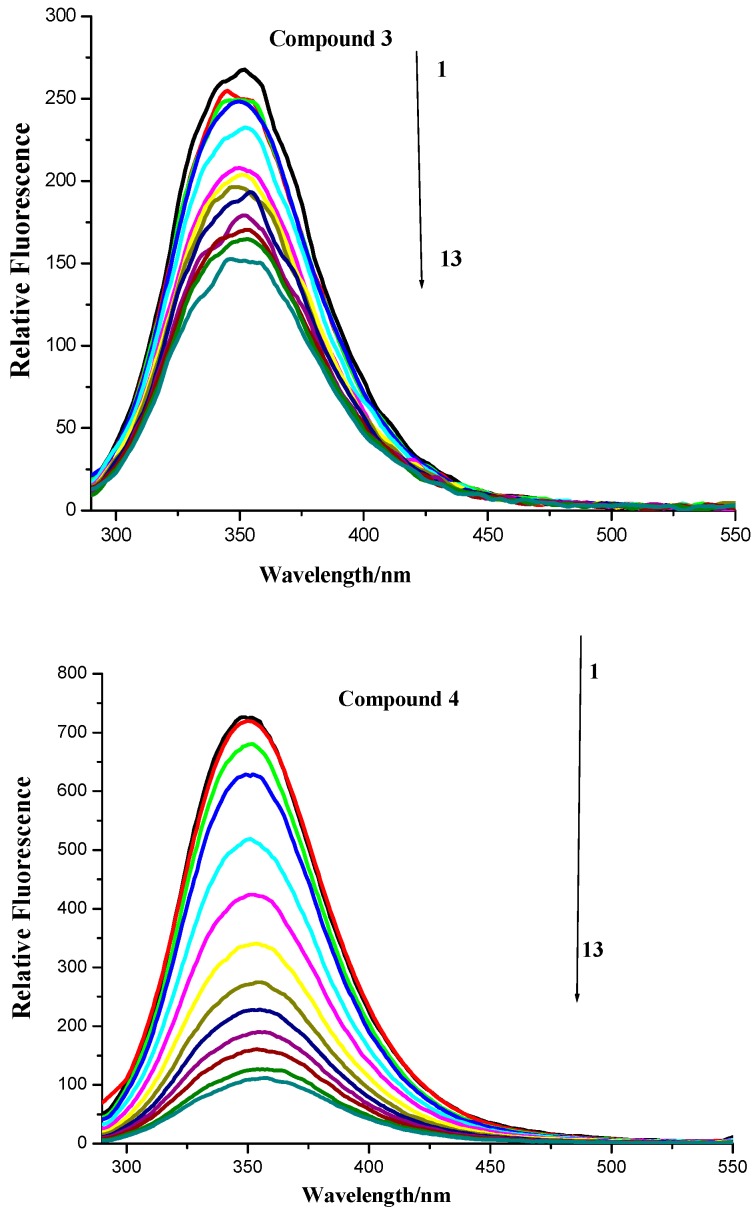
Fluorescence spectroscopy of compounds **1**–**4** and BSA. Numbers 1–13 indicated concentrations of compounds **1**–**4**: 0.0, 0.4 ×10^−6^, 0.8 × 10^−6^, 1.2 × 10^−6^, 2.4 × 10^−6^, 3.6 × 10^−6^, 4.8 × 10^−6^, 6.0 × 10^−6^, 7.2 × 10^−6^, 8.4 × 10^−6^, 9.6 × 10^−6^, 10.8 × 10^−6^ and 12 × 10^−6^ mol∙L^−1^, respectively. BSA concentration applied was 1.04 × 10^−6^ mol∙L^−1^. Scan condition: Ex = 280 nm, Em = 290–550 nm; slits of both Ex and Em of compounds **1**–**3** were 5 nm while those of compound **4** were 5 nm and 10 nm, respectively.

**Figure 5:**
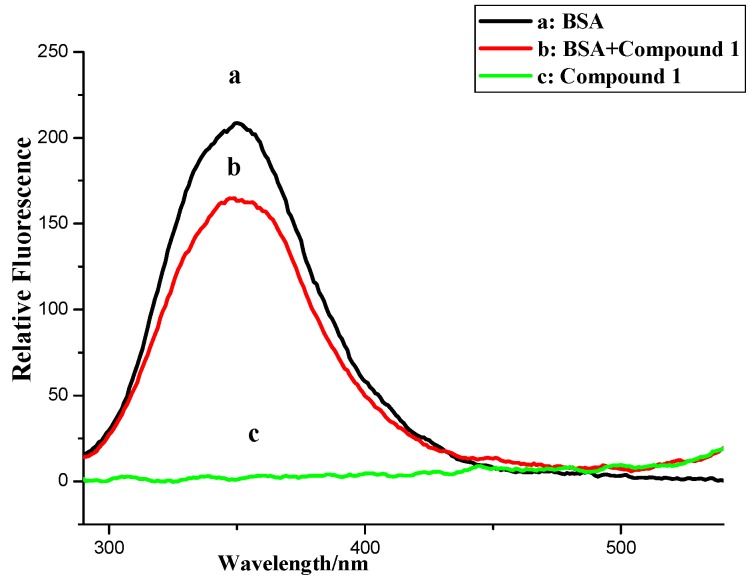

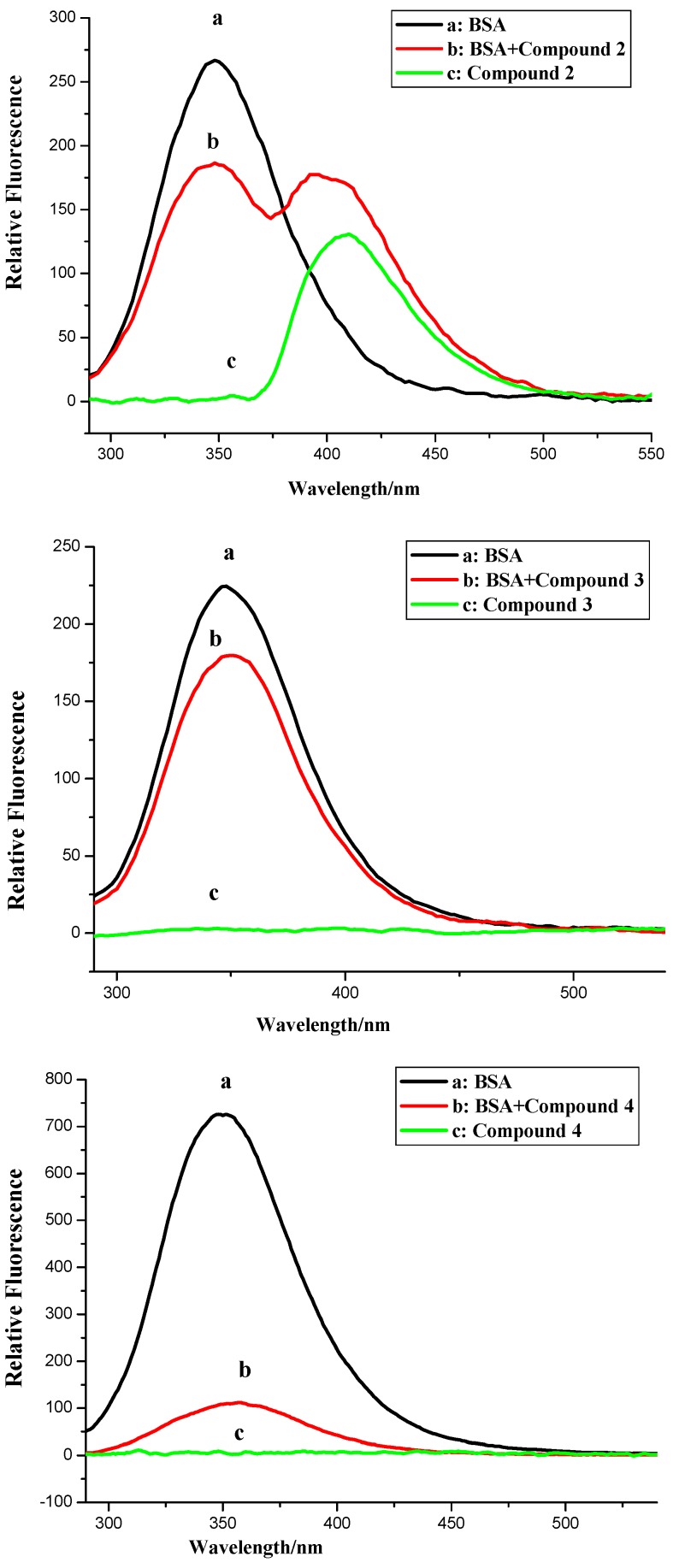
Fuorescence of compounds **1**–**4**, BSA and BSA + compounds **1**–**4**. *c* (compound) = 12 × 10^−6^ mol∙L^−1^; *c* (BSA) = 1.04 × 10^−6^ mol∙L^−1^.

To further investigate the BSA binding properties of naphthalimide-polyamine conjugates, FL spectrometry was applied because the inherent fluorescence of BSA allowed us to examine the compounds’ interaction with BSA. As shown Figure 4 and Figure 5, compounds **1**, **3** and **4** have no emission from 290 to 500 nm while compound **2** had no emission from 290 to 365 nm, which implied compounds **1**–**4** had little effect on the fluorescence analysis. As also shown in Figure 4, the fluorescence intensities of BSA at 350 nm with an excitation wavelength of 280 nm exhibited a remarkable downward trend as the concentrations of compounds **2**–**4** increased. Thus indicated that the possible formation of relevant complexes between compounds **2**–**4** and BSA could influence the secondary structure of the protein that would in turn result in changes in the microenvironment around the tyrosine (Tyr) and tryptophan (Try) residues of BSA [22]. Surprisingly, the fluorescence quenching of BSA by compound **1** was somewhat irregular because of the occurrence of rebounds as the concentrations of compound **1** increased.

**Figure 6:**
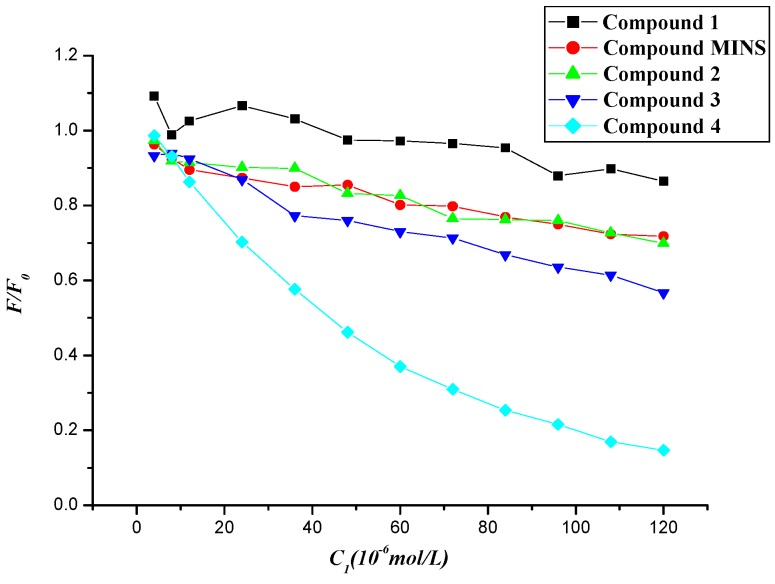
Fluorescence quenching ratio (*F*/*F*_0_) of BSA by compounds **1**–**4** and MINS at room temperatures.

The fluorescence quenching ratio (*F*/*F*_0_) of BSA by compounds **1**–**4** and MINS at room temperatures was then obtained, in which *F*_0_ and *F* are the fluorescence intensities in the absence and presence of quenchers, respectively. In Figure 6, fluorescence quenching capability of compounds **1**–**4** and MINS [20] is depicted in the following order of **4** > **3** > **2** and MINS **>** **1**. Compared to the parent compound MINS, the electron-withdrawn group (-NO_2_) facilitated the fluorescence quenching process, while the presence of electron-donor amino group displayed less impact on the corresponding quenching process.

#### 2.2.2. Fluorescence Quenching Mechanism

Fluorescence quenching can occur by different mechanisms, which are usually classified as either dynamic or static quenching. Dynamic quenching results from the diffusive encounter between the quencher and fluorophore during the lifetime of an excited state, causing the bimolecular quenching constants to increase in value at higher temperatures. Static quenching, however, results from the formation of a ground state complex between the fluorophore and quencher. This concept thus explains the decrease in stabilities and static quenching constants as temperature elevates [23]. In order to draw clarity from the quenching mechanism of the interaction between naphthalimide-polyamine conjugates and BSA, fluorescence quenching tests as described by the Stern-Volmer equation were also carried out at 298, 303 and 310 K, respectively [2,24].

**Figure 7:**
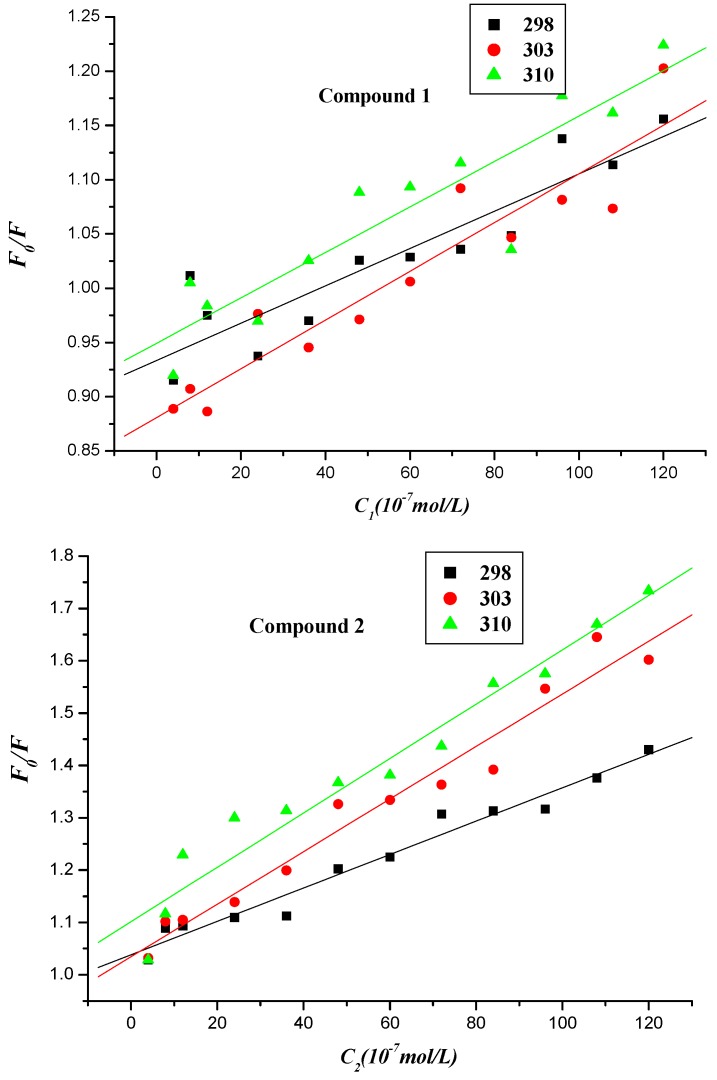

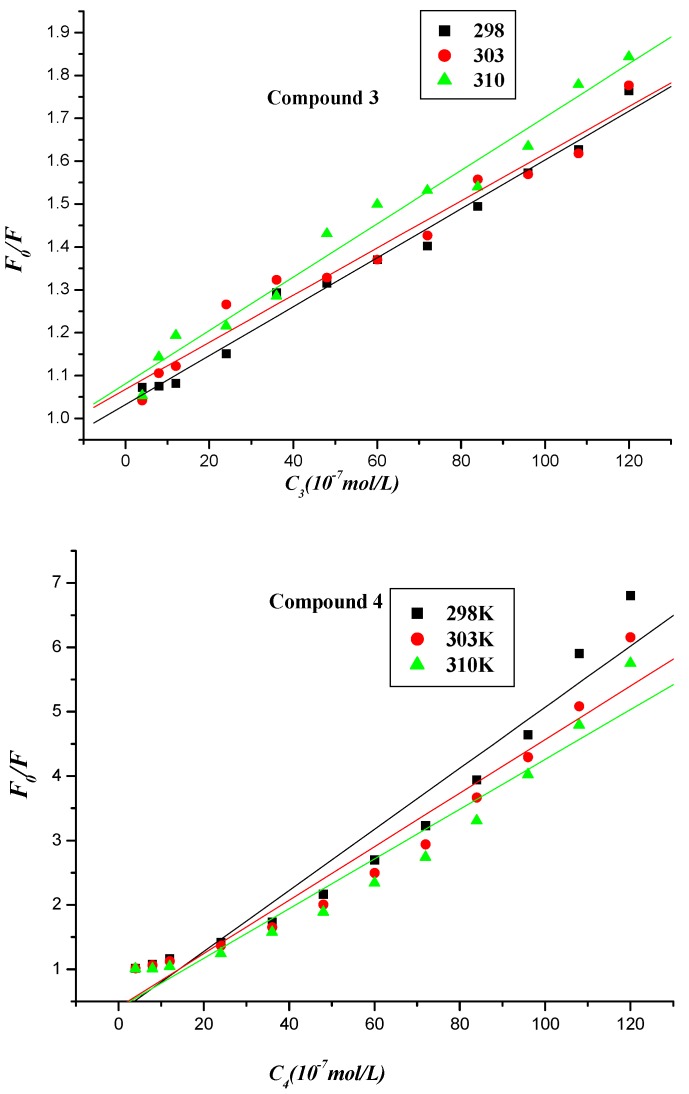
Stern-Volmer linear plot of fluorescence quenching of BSA by compounds **1**–**4** at different temperatures.

The Stern-Volmer equation is the following:

*F*_0_/*F* = 1 + *K*_SV_ × *c* = 1+ *K* × τ_0_*×c* (1)

in which *F*_0_ and *F* are the same as described above. *K_SV_* is the Stern-Volmer quenching constant, [*c*] is the concentration of compounds **1**–**4**, *K_q_* is the biomolecule quenching rate constant, and τ_0_ is the average lifetime of the molecule without any quencher in *Kq* = *K_SV_*/τ_0_, with the average fluorescence lifetime of the biopolymer around 6.0 × 10^−9^ s at 298 K, (Figure S1 and Table S1) [25,26]. The Stern-Volmer plots of *F*_0_/*F* *vs.* [*c*] at the three temperatures were shown in Figure 7, and the calculated *K_SV_* and *K_q_* values are listed in Table 1.

The values of *K_q_* (>10^12^ L∙mol^−1^) were much higher than the diffusion limit for the quenching rate constant (2.000 × 10^10^ L∙mol^−1^) at 298 K, indicating that the fluorescence quenching mechanism of BSA initiated by compounds **1**–**4** was static quenching in nature because the diffusion limit for the quenching rate constant *K_q_* of dynamic quenching of various quenchers with the biopolymer is 2.000 × 10^10^ L∙mol^−1^ [22]. The values of quenching constant *K_SV_* of compounds **1**–**3**, however, generally increased with increasing temperature, which revealed that the interaction mechanisms of BSA initiated by compounds **1**–**3** were not typically static types and they were accompanied by dynamic quenching [27]. It is reported that static quenching constant does not necessarily decrease with increasing temperature, and it sometimes increases or stays constant [28,29,30,31]. Thus, the fluorescence quenching mechanism of BSA triggered by compounds **1**–**3** was overall static. On the contrary, the values of quenching constant *K_SV_* of compound **4** decreased with the increasing temperature, indicating that the fluorescence quenching mechanism of BSA initiated by compound **4** was a typically static type. Moreover, the values of quenching constant *K_SV_* of compounds **1**–**4** and MINS decreased in the following manner: **4** > **3** > **2** > MINS > **1**, implying that the substituent groups affected the fluorescence quenching process of BSA by compounds **1**–**4**, compared to MINS with a naked aromatic ring [20]. Moreover, compound **1**, which has the electron-donor amino group, displayed the least impact on the corresponding quenching process, so it has the smallest quenching constant *K_SV_* value at room temperature. It is well known that the fluorescence of BSA results from Trp and Tyr residues. The fluorescence of BSA came from both Trp and Tyr residues with an excitation wavelength of 280 nm, while only Trp residues were excited at a 295 nm wavelength [32,33]. Therefore, the fluorescence of BSA with or without compounds at 298, 303 and 310 K was also measured at the excitation wavelength of 295 nm (Figures S2–S5 and Table S2).

**Table 1:** Quenching constant of the interaction between compounds **1**–**4** and BSA at different temperatures.

Compound	*T* (K)	*K_sv_* (L∙mol^−1^)	*K_q_* (10^12^ L∙mol^−1^)	*r*
**1**	298 303 310	(1.720 ± 0.243) × 10^4^ (2.250 ± 0.252) × 10^4^ (2.090 ± 0.295) × 10^4^	(2.895 ± 0.409) × 10^12^	0.9133 0.9424 0.9137
**2**	298 303 310	(3.190 ± 0.179) × 10^4^ (5.020 ± 0.304) × 10^4^ (5.200 ± 0.364) × 10^4^	(5.369 ± 0.301) × 10^12^	0.9846 0.9822 0.9763
**3**	298 303 310	(5.700 ± 0.223) × 10^4^ (5.500 ± 0.318) × 10^4^ (6.220 ± 0.304) × 10^4^	(9.594 ± 0.375) × 10^12^	0.9924 0.9837 0.9883
**4**	298 303 310	(4.745 ± 0.354) × 10^5^ (4.159 ± 0.302) × 10^5^ (3.864 ± 0.293) × 10^5^	(7.987 ± 0.596) × 10^13^	0.9735 0.9747 0.9724

A comparison of the BSA fluorescence quenching excited at 280 and 295 nm allows us to evaluate the role of the Trp and Tyr groups in the complex. Indeed, an obviously decreased BSA fluorescence intensity was obtained using 295 nm as the excitation wavelength (Figure S5). In addition, the fluorescence quenching of BSA by compounds **2**–**4** when excited at 280 nm was more significant than at 295 nm. The *K_SV_* variation trend of compounds **2**–**4** excited at 295 nm was consistent with those at 280 nm. However, the distinctive behavior of compound **1** with a higher *K_SV_* value at 295 nm than at 280 nm, might originate from its unusual existence in BSA, as illustrated in subsequent molecular docking experiments. Finally, both the *K_q_* values (>10^12^ L∙mol^−1^) at 280 and 295 nm corroborated that the fluorescence quenching mechanism of BSA initiated by compounds **1**–**4** was a static type.

#### 2.2.3. Interaction Mode between Compounds **1**–**4** and BSA

**Figure 8:**
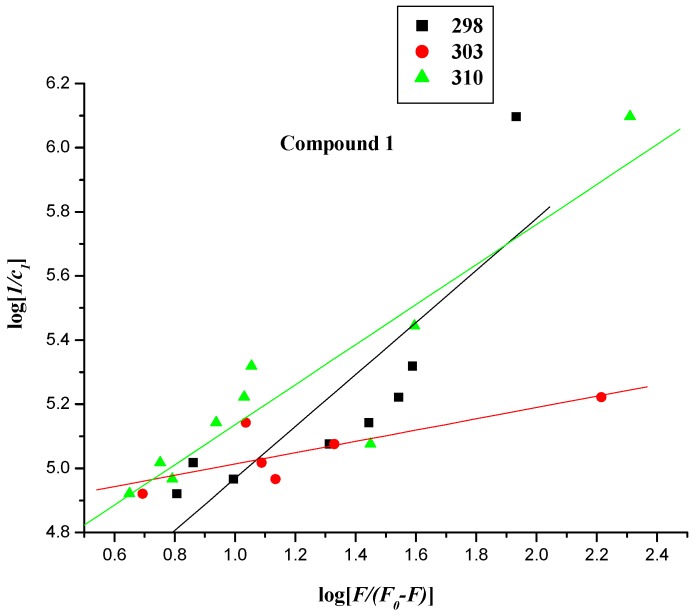

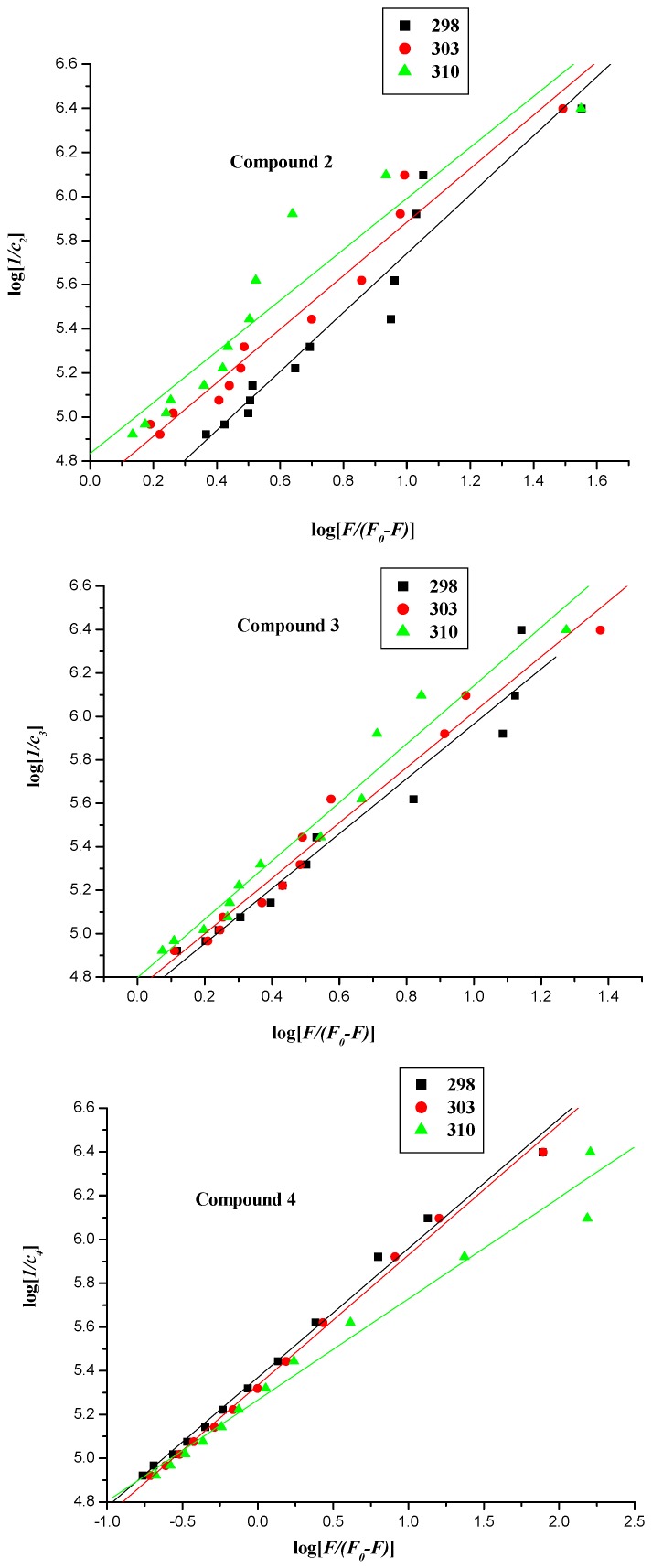
Linear plot of log [1/*c_comp._*] *vs.* log [*F*/(*F*_0_ − *F*)] of the interaction between compounds **1**–**4** and BSA at different temperatures.

For a static quenching process, when small molecules bind independently to a set of equivalent sites in a macromolecule, the binding constant (*K_b_*) for a polyamine conjugate-BSA system can be determined by the following equation [34,35]:

Log [1/*c*] = log [*F*/*(F*_0_ − *F*)] + log *K_b_* (2)

in which *K_b_* denotes the binding constant for the interaction between naphthalimide–BSA. *F*_0_, *F*, and [*c*] have the same definitions as described in Equation (1). The values of *K_b_* were measured from the intercept and slope by plotting log [1/c] against log [*F*/(*F*_0_ − *F*)] (intercept = log *K_b_*) (Figure 8), with the corresponding values of *K_b_* presented in Table 2. The changes in trend of *K_b_* with increasing temperatures were in accordance with *K_SV_*’s dependence on temperature as mentioned above, implying that the binding between naphthalimide and BSA was moderate, and a reversible naphthalimide-BSA complex might have been formed [36]. Experimental data showed that the values of binding constant *K_b_* decreased in a manner similar to that of BSA at room temperature: **4** > **3** > **2** > MINS > **1**, providing further evidence to the effect of substituent groups appending on naphthalimide (Table 2).

**Table 2:** Binding constants and thermodynamic parameters of the interaction between compounds **1–4** and BSA at different temperatures.

Compound	*T* (K)	*K_b_* (L∙mol^−1^)	*∆G°* (L∙mol^−1^)	*∆H°* (kJ∙mol^−1^)	*∆S°* (kJ∙mol^−1^)	*r*
**1**	298	1.438 × 10^4^ ± 1.923	−23.718	52.076	0.2543	0.8464
	303	6.879 × 10^4^ ± 1.217	−28.060	52.076	0.2543	0.8093
	310	3.244 × 10^4^ ± 1.361	−26.771	52.076	0.2543	0.9137
**2**	298	2.541 × 10^4^ ± 1.246	−25.913	91.697	0.932	0.9652
	303	4.680 × 10^4^ ± 1.134	−27.090	91.697	0.932	0.9813
	310	6.823 × 10^4^ ± 1.208	−28.687	91.697	0.932	0.9435
**3**	298	5.040 × 10^4^ ± 1.146	−26.827	14.915	0.140	0.9769
	303	5.567 × 10^4^ ± 1.101	−27.527	14.915	0.140	0.9873
	310	6.281 × 10^4^ ± 1.115	−28.474	14.915	0.140	0.9820
**4**	298	2.341 × 10^5^ ± 1.030	−30.361	−12.221	0.143	0.9961
	303	2.158 × 10^5^ ± 1.022	−30.940	−12.221	0.143	0.9980
	310	1.847 × 10^5^ ± 1.056	−31.254	−12.221	0.143	0.9884

The interactive forces between a small organic molecule and biomacromolecules may include hydrophobic forces, hydrogen bonds, van der Waals forces, and electrostatic interactions. It is assumed that the interaction enthalpy change (∆*H*°) varies significantly within the limited temperature range (entropy change, *∆S°*) studied. Free energy change (∆*G*°) is calculated from van’t Hoff equation:

ln(*K*_2_/*K*_1_) = (1/*T*_1_ − 1/*T*_2_) ∆*H*°/*R* (3)

∆*G*° = − *RT* ln*K =* ∆*H*° *−T*∆*S*° (4)

In Equations (3) and (4), *K* is the binding constant at the corresponding temperature and R is the gas constant. Enthalpy change (∆*H*°) and entropy change (∆*S*°) are calculated from Equations (3) and (4), with the corresponding results listed in Table 2.

Ross *et al*. described the sign and magnitude of thermodynamic parameters associated with the various kinds of interaction, which might take place in protein-associated processes, as characterized below [37]: (a) positive Δ*H* and Δ*S* values are frequently taken as evidence for typical hydrophobic interactions; (b) negative Δ*H* and Δ*S* values arise from van der Waals forces and hydrogen bond formation; (c) positive Δ*S* and negative Δ*H* values are characterized as specific electrostatic interactions between ionic species in aqueous solution. In Table 2, the positive ∆*H*° and ∆*S*° values of compounds **1**–**3** indicated that hydrophobic interactions played a dominant role in the interactions between naphthalimides and BSA [37]. However, the negative ∆*H*° and positive ∆*S*° values of compound **4** showed that the electrostatic interaction played a dominant part in the interactions between polyamine conjugate **4** and BSA, which has two strong electron-withdrawing nitro-group and displayed the strongest impact on the corresponding binding process [37]. These results confirmed that substituent groups affected the interactive mode between naphthalimides and BSA.

The binding constant (*K_b_*) and thermodynamic parameters of the interaction between compounds **1**–**4** and BSA were also assayed at an excited wavelength of 295 nm (Figure S4 and Table S3). The results showed the mode of interaction of BSA with compounds **1**–**4** was consistent with that at 280 nm. Surprisingly, the *K_b_* values at 295 nm were distorted, making it difficult to see a pattern with related substituents on aromatic rings, compared to those at 280 nm.

#### 2.2.4. Synchronous Fluorescence Spectroscopy

The synchronous fluorescence spectroscopy of BSA can provide characteristic information of tyrosine or tryptophan residues when wavelength interval (∆λ) between excitation wave length and emission wavelength is at 15 or 60 nm [38].

**Figure 9:**
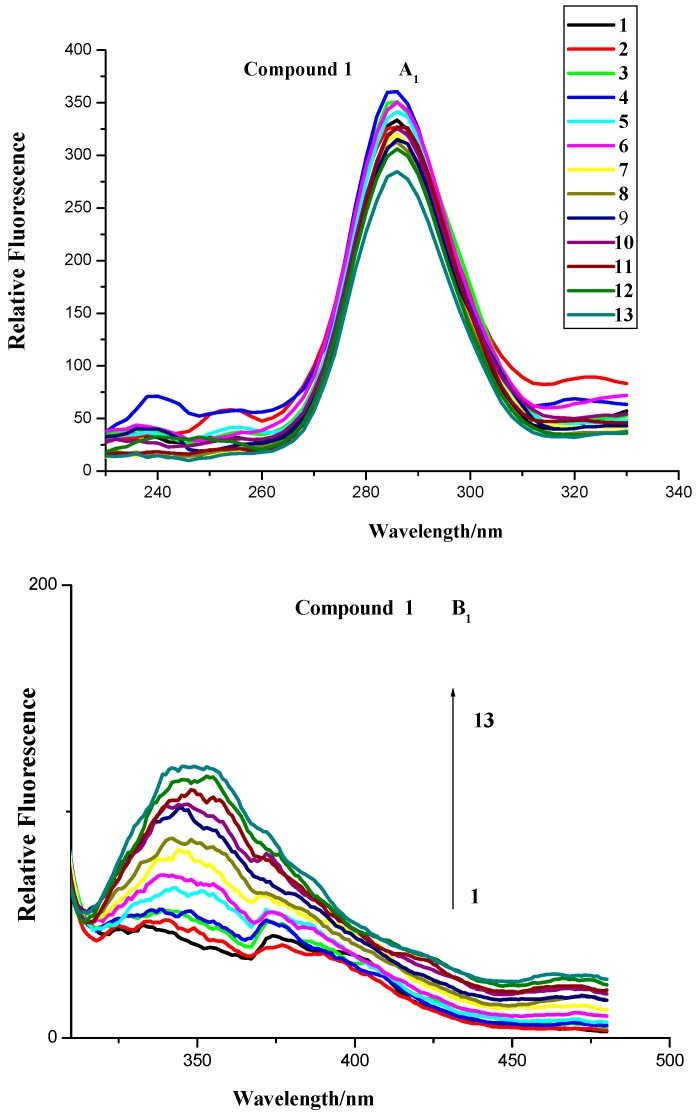

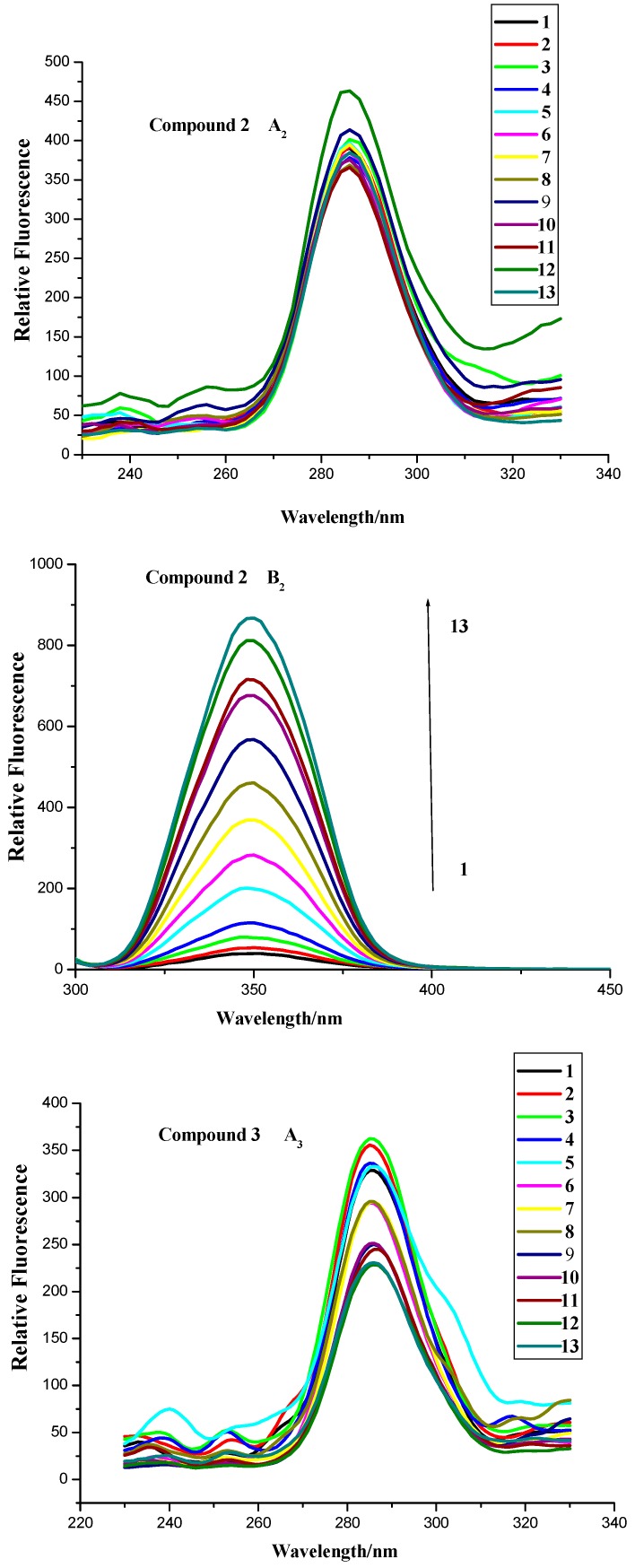

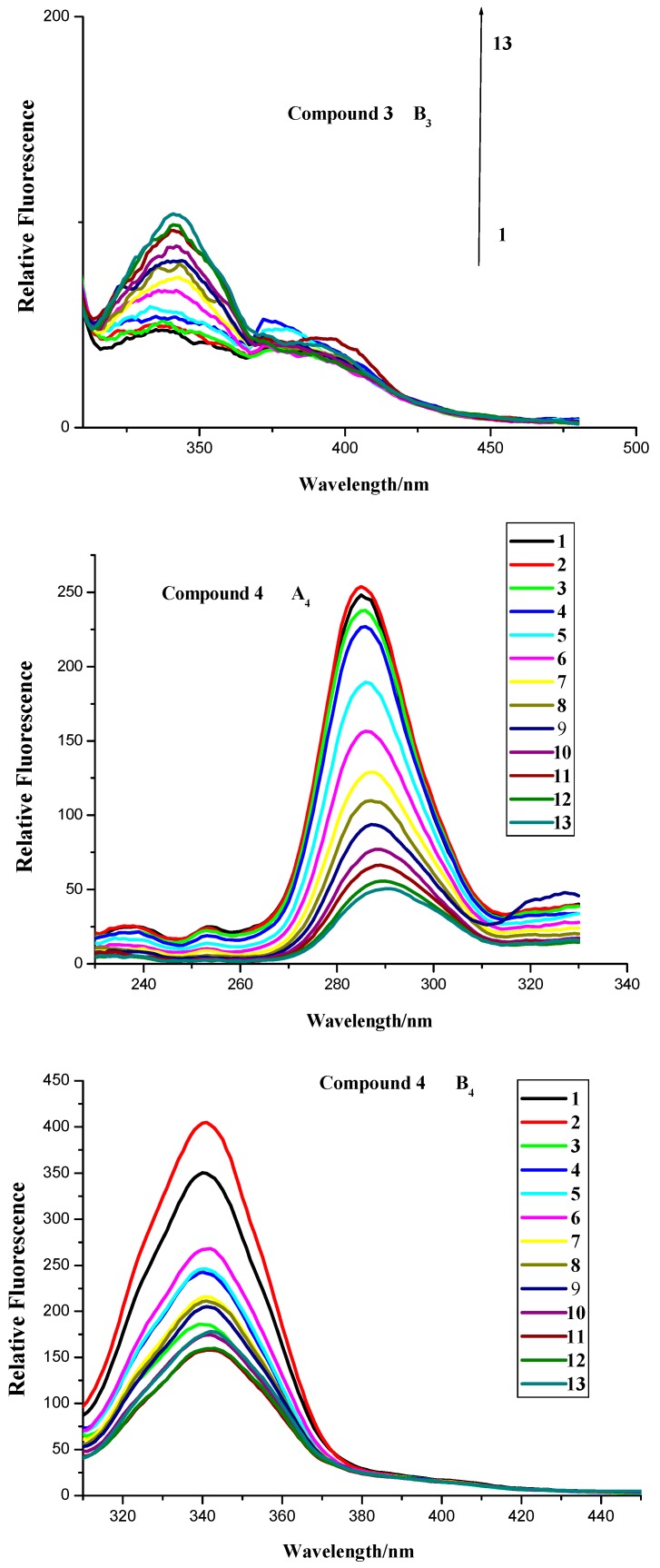
Synchronous fluorescence spectra of compounds-BSA system. (A: Δλ = 15 nm, B: Δλ = 60 nm). The concentrations of BSA and compounds were the same as those in Figure 4.

When compounds **1**–**3** were added to the BSA solution, fluorescence emission intensities of tyrosine residues increased or were quenched irregularly, and the corresponding wavelengths had no obvious shift (Figure 9A_1–4_). The fluorescence emission of tyrosine residues, however, was remarkably quenched as the concentration of compound **4** increased. In contrast, the addition of compounds **1**–**3** remarkably increased the fluorescence emission intensities of tryptophan residues with the irregular quenching caused by compound **4** (Figure 9B_1–4_). Compounds **1** and **3** resulted in a respective red shift of 16 and 6 nm, while compound **2** and **4** had no impact on the wavelength, which indicated that the hydrophobicity around the tryptophan residues was not increased [39].

These results suggested that compounds **1**–**4** were not easily localized in the hydrophobic cavity to bind with tyrosine and tryptophan residues. Thus these compounds had only a slight influence on the conformation of tyrosine residues in the subdomain of BSA. The formed compounds **1**–**4-**BSA complexes lowered the hydrophobicity of tryptophan residues and in turn caused the structures to be less compact.

#### 2.2.5. Three-Dimensional Fluorescence Spectroscopy Analysis

**Figure 10:**
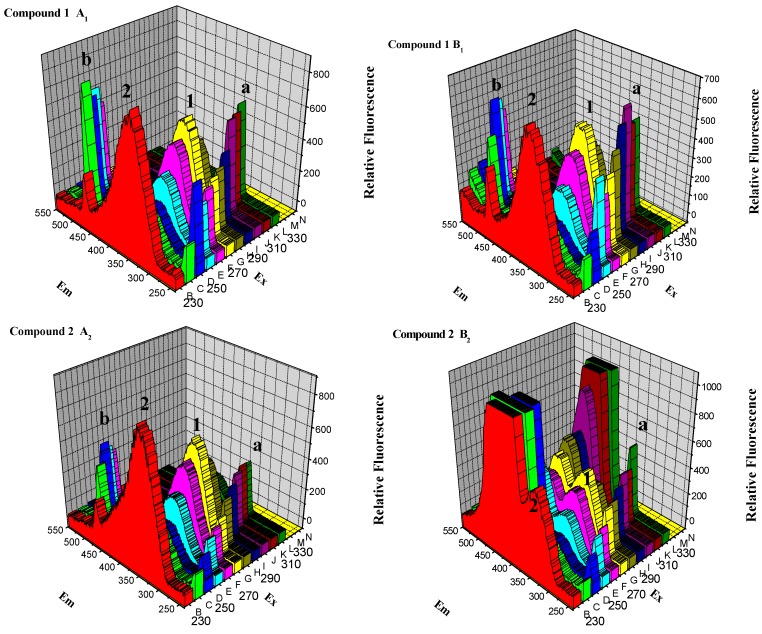

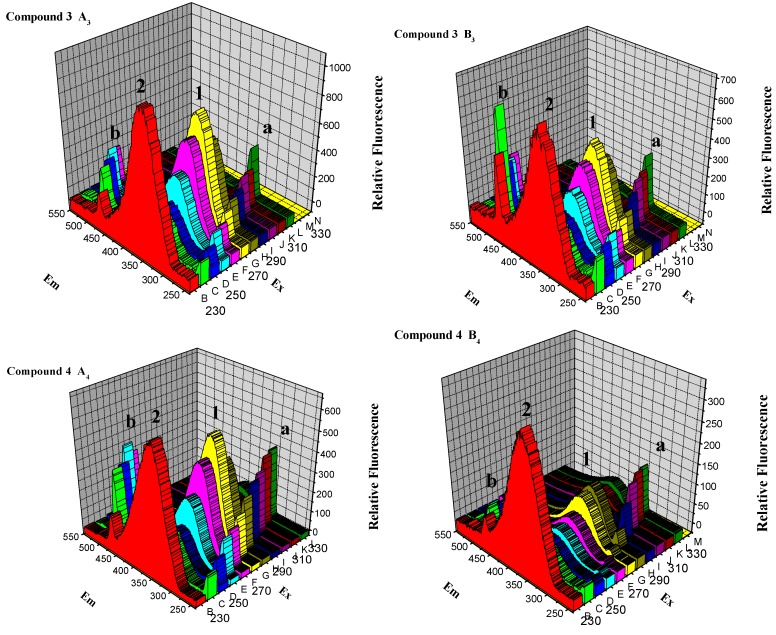
Three-dimensional fluorescence spectra of BSA in the absence (**A**) and presence (**B**) of compounds **1**–**4**. Conditions: *c* (BSA) = 1.04 × 10^−6^ mol∙L^−1^, *c* (compounds **1**–**4**) = 12.0 × 10^−6^ mol∙L^–1^.

Three-dimensional fluorescence spectra of BSA in the absence and presence of compounds **1**–**4** were investigated. Figure 10_A1–A4_ depict the three-dimensional fluorescence spectra of BSA in the absence of compounds **1**–**4**. Four peaks were found: peak a, peak b, peak 1 and peak 2, respectively. Peak a is the Rayleigh scattering peak (λ_ex_ = λ_em_), and peak b is the second-order scattering peak (2λ_ex_ = λ_em_) [40,41]. Peak 1 (λ_ex_ = 280 nm, λ_em_ is about 350 nm), the primary fluorescence peak, represents the spectral characters of tyrosine and tryptophan residues [42,43,44]. Peak 2 (λ_ex_ = 230 nm, λ_em_ is around 350 nm) accounts for the fluorescence may be from excitation of indole into S2 and subsequent emission from S1, undergoing intersystem crossing in BSA.

As shown in Figure 10B_1–4_, the presence of compounds **1**–**4** reduced the fluorescence intensity of peak 1 by 15.73%, 24.50%, 44.11% and 81.55%, respectively, implying the altered microenvironments of tyrosine and tryptophan residues [45]. The fluorescence intensity of peak 2 was also reduced by 25.13%, 40.01%, 37.32% and 55.76%, respectively, indicating that peptide strand structures of BSA were changed as well. These results supported that the interaction between compounds **1**–**4** and BSA triggered slightly microenvironmental and conformational alterations in BSA [46,47]. Besides, the reduced fluorescence intensity of peak 1 with the order of **4** > **3** > **2** > **MINS** > **1** at room temperature provided further evidence of the effect of substituent groups.

### 2.3. CD Measurement

**Figure 11:**
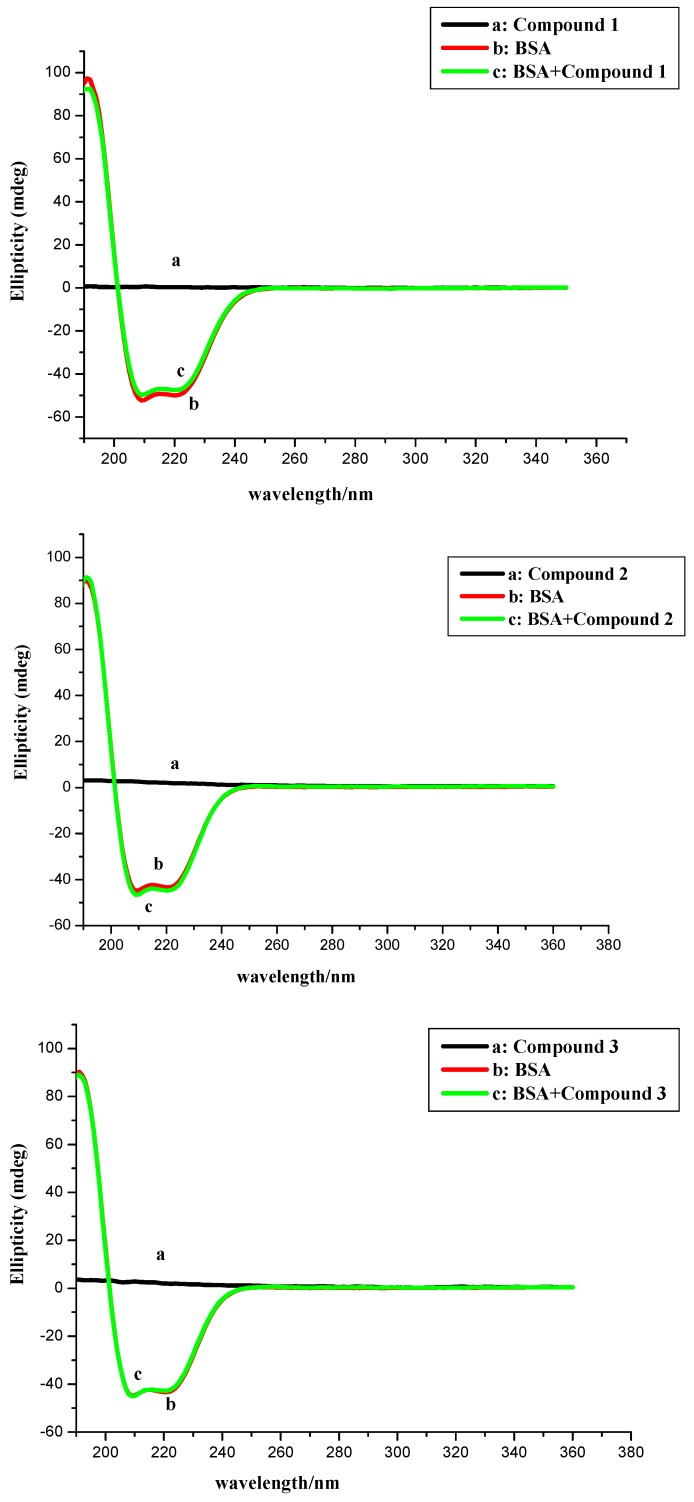

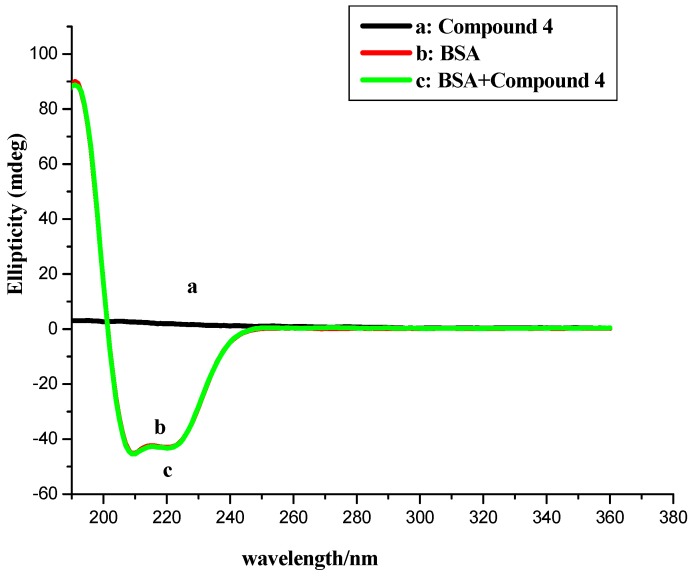
CD spectra of BSA in the presence of the compounds. Conditions: *c* (BSA): 40.0 × 10^–6^ mol∙L^–1^; *c* (conpounds): 0, 120.0 × 10^–6^ mol∙L^–1^.

Circular dichroism (CD) is one of the most valuable methods for examining conformational alterations in protein upon interaction with exogenous substances [48,49,50]. Thus, in order to obtain an insight into the structure of BSA complex, CD spectral study was carried out to observe the conformational change of BSA in the presence of compounds **1**–**4**. The CD spectral study was carried out in PBS (pH = 7.4) buffer solution (Figure 11).

The CD spectra of free BSA displayed two negative bands at 209 and 220 nm, characteristics of the α-helix structure [51,52]. The CD spectra of BSA in presence and absence of compounds **1**–**4** were similar in shape, indicating that the structure of BSA was predominantly α-helix. In addition, the CD signal of BSA insignificantly decreased without any noteworthy wavelength shift in the presence of compounds **1**–**4** at the molar ratio of BSA to compounds 1:3.

From these results, it could be inferred that compounds **1**–**4** caused weak conformational changes in BSA without losing the original helical stability. In addition, protein conformational analysis based on CD data reported that the free BSA had a high α-helical content of 65.5%, antiparallel of 3.4%, parallel of 3.4%, turn of 12.4%, and random coil of 16.1% (Table 3). Upon addition of compounds **1**–**4**, alterations of BSA conformation were not obvious as their respective values are within the typical margin of error, which implied polyamine conjugates caused little protein unfolding in the secondary structure of BSA and meant that compounds **1**–**4** could maintain the stability of the protein conformation [13]. These results were further supported by subsequent thermal stability and molecular docking experiments.

**Table 3:** Secondary structure of compounds **1**–**4-**BSA complex (CD Spectra) at pH 7.4 as calculated by CCDN software.

Compounds 1–4 Concentration (μM)	α-Helix	Antiparallel	Parallel	β-Turn	Random Coil	Total Sum
Free BSA	65.3%	3.4%	3.4%	12.4%	16.1%	100.5%
compound **1**-BSA	67.8%	3.1%	3.1%	12.1%	15.0%	101.1%
compound **2**-BSA	66.8%	3.2%	3.2%	12.2%	15.4%	100.9%
compound **3**-BSA	61.0%	3.8%	3.9%	13.0%	17.9%	99.6%
compound **4**-BSA	61.7%	3.7%	3.8%	12.9%	17.6%	99.7%

**Figure 12:**
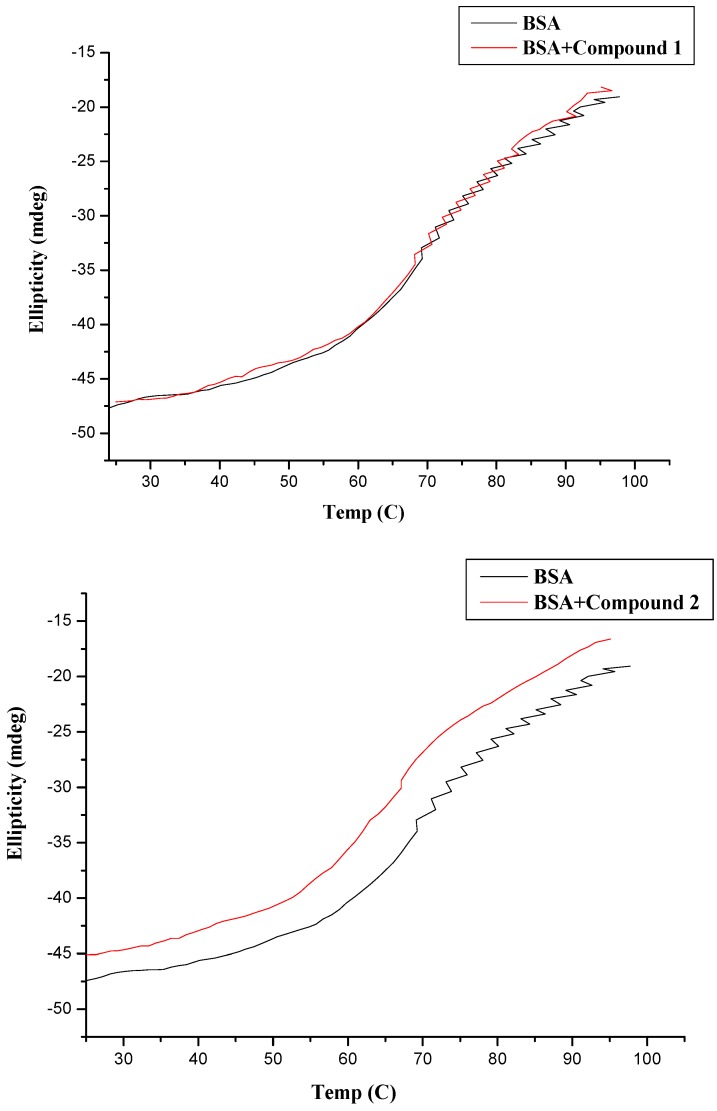

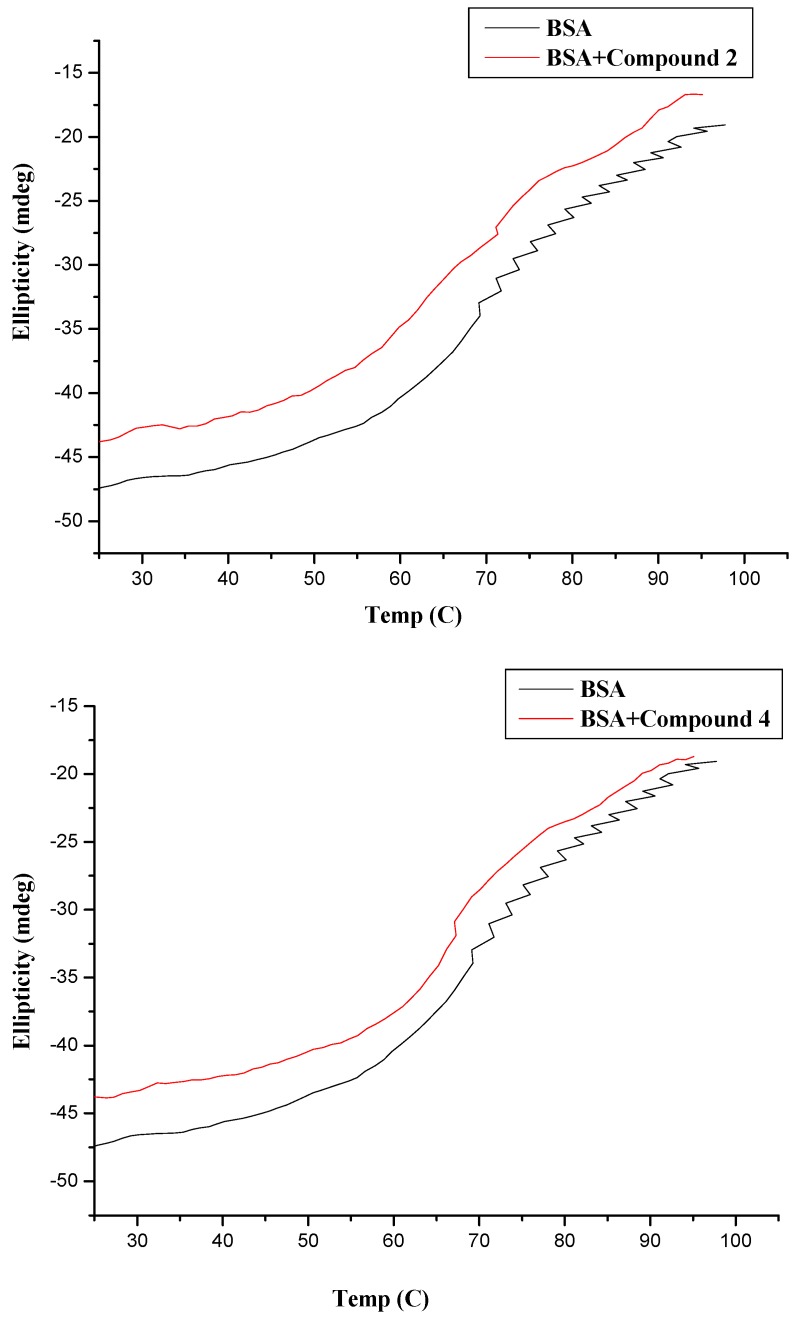
Thermal denaturation of 40 × 10^−6^ mol∙L^–1^ BSA in the absence and presence of 120 × 10^−6^ mol∙L^–1^ compounds at pH = 7.4.

Based on the facts mentioned above, we investigated the thermal stability of the protein as a consequence of binding. Thermal stability curves for compounds-BSA complex and BSA were plotted from 20 °C to 95 °C, as shown in Figure 12. The figure showed no obvious increase in melting temperature (*T_m_*) between BSA alone and the compounds-BSA complex. The slight change in the melting temperature (*T_m_*) of BSA implied that the presence of compounds **1**–**4** did not enhance the thermal stability of BSA and the binding of compounds with BSA resulted in weak structural alterations [51,53].

### 2.4. Docking Analysis

**Figure 13:**
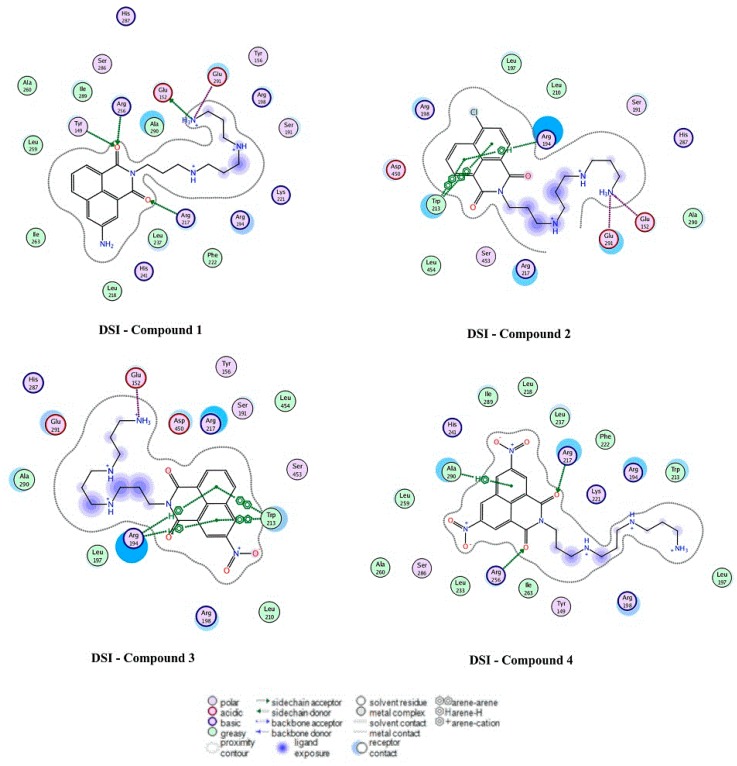
Docking experiment results showing the 2D diagrams of the binding modes of the complex DSI-compounds **1**–**4**. Polar and non-polar residues were represented in red and green circles, respectively. H-bond acceptors or donors were shown in green-dotted arrows. Electrostatic interactions from ion pairs were presented in red-dotted lines. π-π and π-H interactions were represented in green-dotted lines.

The 2-dimensional (2D) and corresponding 3-dimensional (3D) diagrams of the best binding mode of each system were plotted. Results showed that the DSI binding site, with regards to compounds **1**–**3**, that the –COO^−^ of Glu152 was keen on forming ion pairs with the –NH_3_^+^ group (–COO^–^∙∙∙–NH_3_^+^) from the compounds, thus forming a strong electrostatic interaction between the two groups. Additionally, in each of the compounds **2** and **3**, the guanidinium group-π interaction was found between Arg194 of BSA protein and the compounds’ respective conjugate rings (Figure 13 and Table 4), an interaction that was also important in other proteins [54]. Moreover, the π-π interaction between the aromatic ring of Trp213 from BSA and the molecule’s conjugate ring also contributed greatly to the binding of BSA and compounds **2** and **3**, respectively. For compound **4**, two distinct hydrogen bonds were found between the carbonyl groups of the ligand and the -NH_2_ groups of Arg217 and Arg256; moreover, the C-H-π interaction was also identified between Ala290 and its conjugate ring. Corresponding binding free energy was abided in the following order: |*∆G* (compound **4**)| > |*∆G* (compound **1**)| > |*∆G* (compound **3**)| > |*∆G* (compound **2**)|, which might help explain the unique property of compound **1** observed at 295 nm.

**Figure 14:**
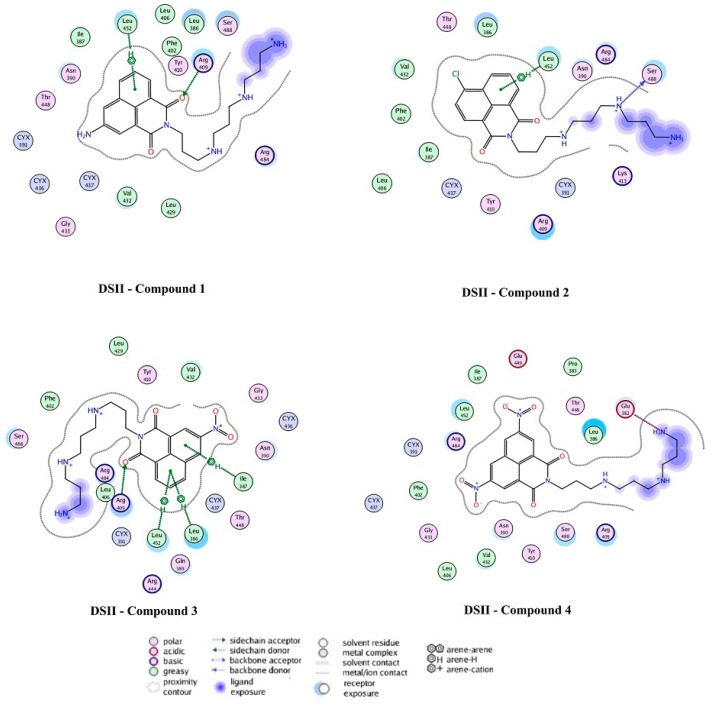
Docking experiment results showing the 2D diagrams of the binding modes of the complex DSII-compounds **1**–**4**. Polar and nonpolar residues were represented in the red and green circles, respectively. H-bond acceptors or donors were shown in green-dotted arrows. Electrostatic interactions from ion pairs were presented in red-dotted lines. π-π and π-H interactions were represented in green-dotted lines.

At the DSII binding site, there was a π-CH_3_ hyperconjugation effect between the –CH_3_ group of Leu452 and the conjugate ring of each of the compounds **1**–**3**. In addition, a similar interaction brought by Ile387 and Leu386 could be seen in compound **3**. As for DSII-compound **4** complex, a strong electrostatic interaction was formed between the –COO^–^ group of Glu382 and the –NH_3_^+^ group of the ligand. At the sites where the affinity energy was only slightly strengthened by electrostatic interaction, polar residues Arg484, Glu449, Thr448, Ser488, Tyr410 and Asn390 would form a cavity to localize the ligand. The corresponding binding free energies could be ordered in the following manner: |*∆G* (compound **4**)| > |*∆G* (compound **3**)| > |*∆G* (compound **2**)| > |*∆G* (compound **1**)|, which is consistent with experimental data at 298 K when an excitation wavelength 280 nm was used (Figure 14 and Table 4). It was worth noting that the ligands (except compound **1**) were more likely to stay at the DSII than at DSI site. In other words, the binding sites of compounds **2**–**4** in BSA were the DSII ones, but compound **1** had not much selectivity to reside in the DSII or DSI site. What might account for this phenomenon was the exposed tail chain of the ligand to the outside of the protein at the DSII site, causing the polar solvent to reinforce the binding free energy (Figure 13). Interestingly, Tyr (other than Trp) residues in the favorable DSII cavity existed around the ligand. Indeed, the spectral data excited at 280 nm matched much better with the molecular docking results, implying the diverse role of Tyr and Trp residues in the detection of BSA-compounds interaction.

**Table 4:** Results for the affinity ∆*G* scoring of molecular docking for BSA and compounds **1**–**4** (DSII refer to the main binding regions within domains II and III, named Sudlow’s sites).

System	Affinity *∆G* Scoring	System	Affinity *∆G* Scoring
DSI–Compound **1**	–6.18	DSII–Compound **1**	–6.28
DSI–Compound **2**	–5.12	DSII–Compound **2**	–6.45
DSI–Compound **3**	–5.39	DSII–Compound **3**	–7.41
DSI–Compound **4**	–7.45	DSII–Compound **4**	–8.49

## 3. Experimental Section

### 3.1. Apparatus

UV-Vis absorption spectra were measured on a Unicam UV 500 spectrophotometer (Beijing, China) through a 1.0 cm cell. Fluorescence measurements were obtained from a Cary Eclipse spectrofluorimeter (Shanghai, China). The fluorescence lifetime of BSA was determined by a FLS980E-S1S1-tm Fluorescence Lifetime Spectrometer (Edinburgh, UK). Circular dichroism spectrum measurements were recorded from a Modle 420 SF (Lake Wood, NJ, USA) automatic recording spectrophotometer through a 1 mm quartz cell.

### 3.2. Materials

Naphthalimide-polyamine conjugates **1**–**4** were prepared previously [18]. The solutions were either prepared with Tris–HCl buffer solution (2.00 × 10^–5^ mol∙L^–1^ for UV and FL) or with phosphate buffer saline (PBS, pH = 7.4) buffer solution (2.00 × 10^–4^ mol∙L^–1^ for CD), and stored at 4 °C. BSA (Sino-American Biotechnology Company, Beijing, China) was used without further purification. And its stock solution, was either prepared by dissolving an appropriate amount of BSA in Tris-HCl (pH = 7.4) buffer solution (2.60 × 10^–5^ mol∙L^–1^ for UV and FL) or mixed with PBS (pH = 7.4) buffer solution (2.00 × 10^–4^ mol∙L^–1^ for CD), and stored at 4 °C.

### 3.3. Procedures

#### 3.3.1. UV-Vis Measurements

A solution of BSA (0.2 mL, 2.60 × 10^−5^ mol∙L^−1^) in Tris-HCl (pH = 7.4) was mixed with 0.0, 0.10, 0.20, 0.30, 0.60, 0.90, 1.20, 1.50, 1.80, 2.10, 2.40, 2.70 and 3.00 mL of each compounds **1**–**4** (2.0 × 10^–5^ mol∙L^−1^), respectively. The mixture was also diluted to 5 mL with Tris-HCl (pH = 7.4). Thus, the final concentrations of samples were 0.0, 0.4, 0.8, 1.2., 2.4, 3.6, 4.8, 6.0, 7.2, 8.4, 9.6, 10.8 and 12.0 × 10^–6^ mol∙L^−1^, respectively. One sample that contained only BSA (1.04 × 10^–6^ mol∙L^−1^) served as control, while others contained different concentrations of compounds **1**–**4** but had the same concentration of BSA. All of the solutions were shaken for 30 min at room temperature.

#### 3.3.2. Fluorescence Measurement

##### Interaction of Compounds **1**–**4** with BSA

The procedure for the preparation of samples was the same as that of UV-Vis samples. Fluorescence wavelengths and intensity areas of samples were measured at 298, 303 and 310 K in the wavelength range of 290–550 nm or 305–580 nm, with the exciting wavelength at 280 nm or 295 nm and the λ_em_ is about 350 nm.

##### Synchronous and Three-Dimensional Fluorescence Spectroscopy

Synchronous fluorescence spectroscopy of compounds **1**, **3** and **4** was obtained with wavelength ranging from 230 to 320 nm (∆λ = 15 nm) and from 290 to 480 nm (∆λ = 60 nm) with the emission slit width of 10 nm. As for compound **2**, the emission slit width was 10 nm for the measurement from 230 to 320 nm, and 5 nm for the measurement from 290 to 480 nm. Three-dimensional fluorescence spectroscopy was conducted under the following conditions: the emission wavelength was recorded from 240 to 550 nm, and the initial excitation wavelength was set at 230 nm with increments of 10 nm.

#### 3.3.3. Fluorescence Lifetime Measurement

For lifetime measurements at the excitation wavelength of 295 nm, a laser, pulse width 0.871 ns, was used to excite the BSA (1.04 × 10^–6^ mol∙L^–1^). The fluorescence decays were deconvoluted and fitted using standard software to obtain lifetimes. The averaged fluorescence lifetime was calculated according to [55].

#### 3.3.4. CD Measurement

A solution of BSA (2 mL, 2.00 × 10^−4^mol∙L^−1^) in PBS (pH = 7.4) was mixed with 0 and 3.00 mL of compounds **1**–**4** (2.00 × 10^−4^ mol∙L^–1^), respectively. The mixture was diluted to 5 mL with PBS (pH = 7.4). Thus, samples were prepared in concentrations of 0.0 and 120.0 × 10^−6^ mol∙L^−1^, respectively. One sample that contained only BSA (40 × 10^–6^ mol∙L^−1^) served as control, while others contained different concentrations of compounds **1**–**4** but had the same concentration of BSA. All of above solutions were shaken for 30 min at room temperature. Temperature scans were carried out between 20 °C and 95 °C, with a scan rate of 1 °C/s and a path length cuvette of 1 mm. The wavelength used was 222 nm.

#### 3.3.5. Molecular Modeling Study

To fully understand the mechanism of the interaction between compounds **1**–**4** and BSA (PDB ID: 4JK4), molecular modeling study *in silico* was performed by using the MOE 2013 Program [56,57,58]. Protonation states of ionizable residues were determined at pH = 7.4. Ligand conformation was identified with minimization. Herein, both the protein and compounds were treated with Amber99SB [59,60,61] with the Merck molecular force field set to 94 s parameters [62]. Two enzymatic drug sites (DSI and DSII) served as docking sites, and eight systems were generated, with each system analyzed in great detail. Affinity *∆G* and London *∆G* scoring were used to estimate the binding mode. 500 random conformations were produced, with the 10 most optimal used for analysis.

## 4. Conclusions

The interaction between compounds **1**–**4** and BSA was studied by spectroscopic methods and molecular docking experiments. The presence of substituent groups on the naphthalimide backbone resulted in a series of changes in the spectral characteristics of the compounds-BSA complexes, with the exceptional behavior of the amino group on the aromatic ring of compound **1** being particularly notable. From the experimental results, it could be affirmed that compounds **1**–**4** were able to effectively combine with BSA and the quenching mechanism was static. Furthermore, the fluorescence quenching data measured at different temperatures (298, 303 and 310 K) suggested that the type of force acting in the compounds **1**–**3-**BSA interaction was mainly hydrophobic in nature. However, synchronous and 3-D fluorescence spectral data provided some evidence for the presence of these hydrophobic interactions between compounds **1**–**4** and BSA. Moreover, compounds **1**–**4** had only weak influences on conformational changes of BSA throughout the BSA binding process. Molecular mechanism calculations were carried out and the calculated results suggested that enzymatic drug site II had little priority over enzymatic drug site I, as the calculated binding-affinity scoring of DSI-compound **1** and DSII-compound **1** was almost the same in value. This might support the abnormal spectral characteristic of compound **1-**BSA complex. However, the DSII in BSA protein was more beneficial than its DSI counterpart in terms of binding with compounds **2**–**4** because the calculated binding affinity scorings of DSI-compounds **2**–**4** were significantly higher than those of DSII-compounds **2**–**4**. Based on simulation results of DSII-compounds **1**–**4**, the main force that governed the complex of compounds **1**–**3-**BSA protein was hydrophobic interaction, as seen by π∙∙∙–CH_3_ hyper- conjugation between the conjugate ring of each of the compounds **1** and **2** and the –CH_3_ group of Leu452 from BSA, as well as between compound **3** and Leu452, Ile387 and Leu386 from BSA. The hydrophobic nature of interactions was confirmed by thermodynamic experimental results that showed positive Δ*H* and Δ*S* values. The strong electrostatic interactions formed from the charged interaction between the –NH_3_^+^ group of compound **4** and –COO^–^ group of Glu382, and between the compound’s charged conjugate ring and the charged cavity constructed by the residues (Arg484, Glu449 *et al.*) of BSA protein were the main contributors to the binding of compound **4** and the BSA protein, findings supported by thermodynamic results that reported a positive Δ*S* (0.143 kJ∙mol^−1^) and negative Δ*H* (−12.221 kJ∙mol^−1^) values, according to Ross *et al.* [37].

## Supplementary Materials

Supplementary materials can be accessed at: http://www.mdpi.com/1420-3049/20/09/16491/s1.
