# Supplementary material for: Spectroscopic Study on the Interaction between Naphthalimide-Polyamine Conjugates and Bovine Serum Albumin (BSA)
Source: Molecules. 2015 Sep 11;20(9):16491–523. doi: 10.3390/molecules200916491 (PMC6332075; doi:10.3390/molecules200916491)
Supplement: Supplementary file 1 [file molecules-20-16491-s001.pdf]

Supporting Information

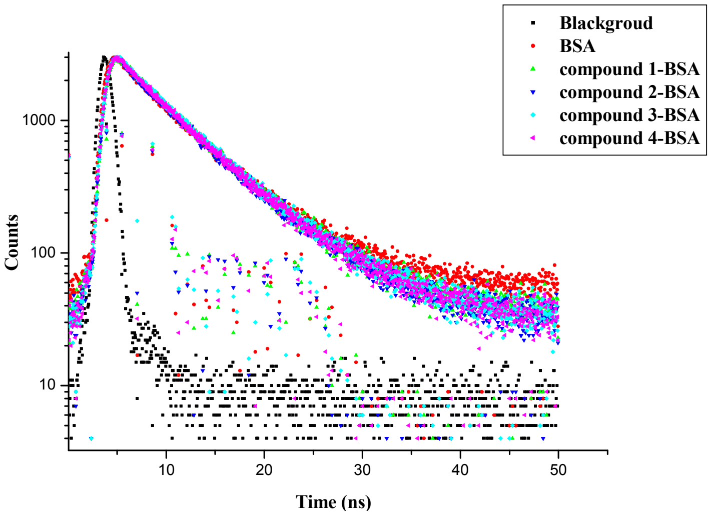

Figure S1. The fluorescence lifetimes of the BSA and compounds **1**–**4**-BSA complex; c (BSA) = 1.04 × 10^{-6} mol·L^{-1}; c (compounds **1**–**4**) = 12.0 × 10^{-6} mol·L^{-1}.

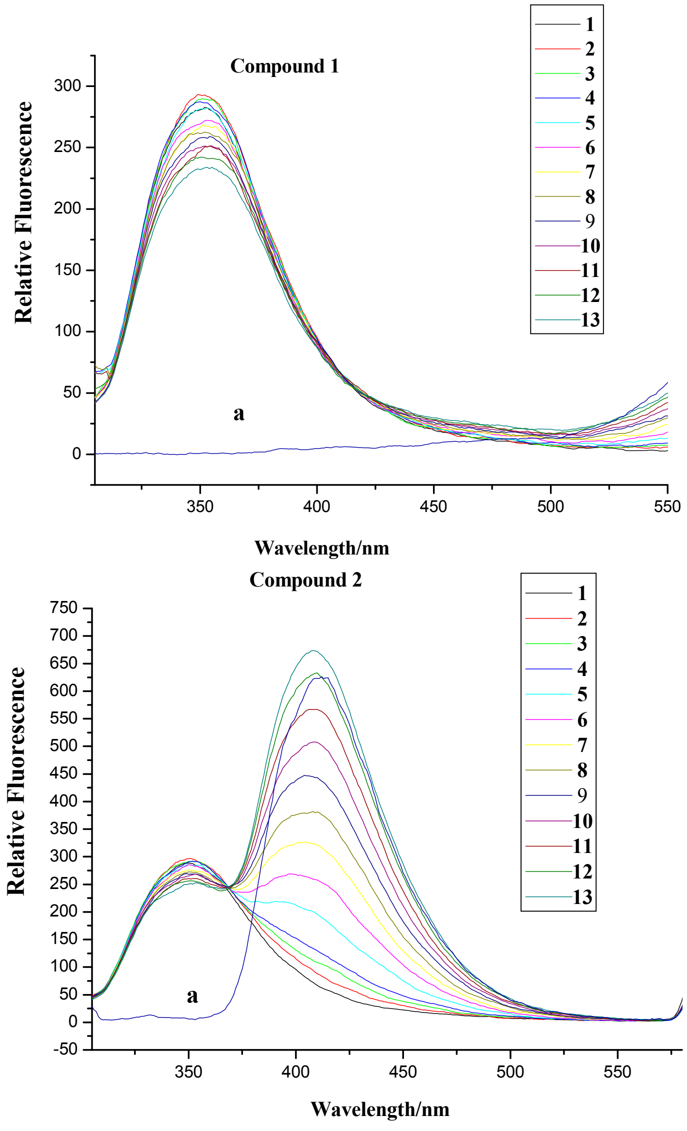

Figure S2. *Cont.*

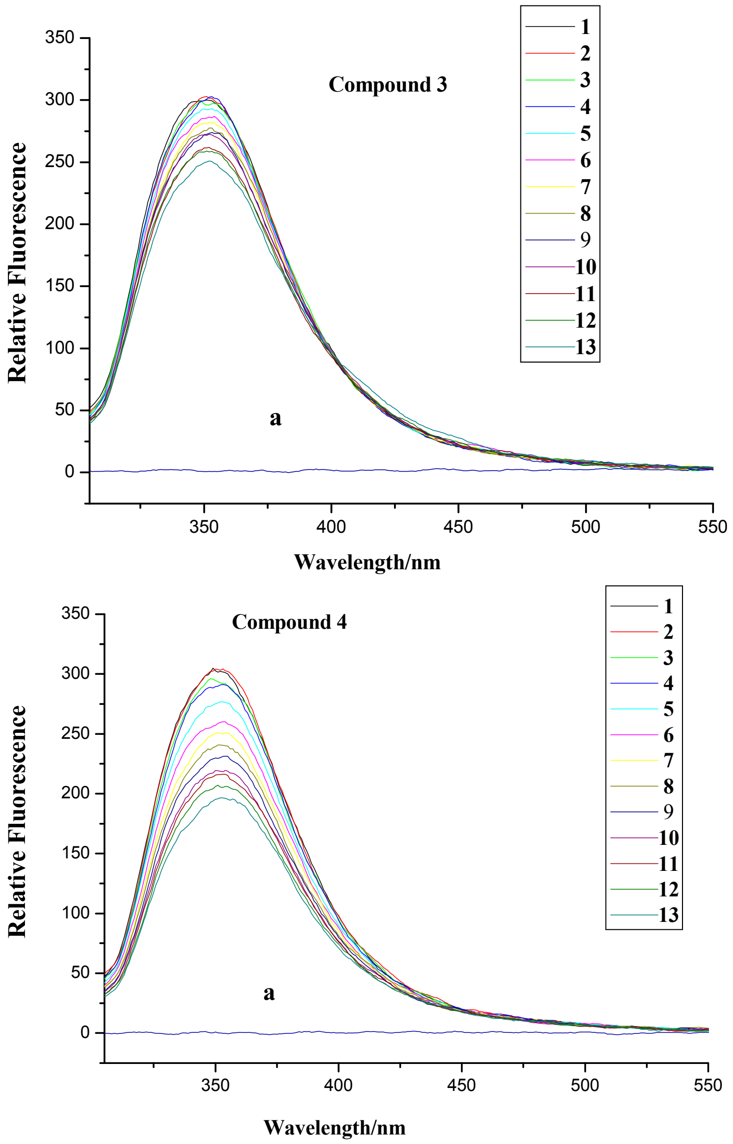

Figure S2. Fluorescence spectroscopy of compounds **1–4** and BSA. Numbers 1–13 indicated concentrations of compounds **1–4**: 0.0, 0.4×10^{-6}, 0.8×10^{-6}, 1.2×10^{-6}, 2.4×10^{-6}, 3.6×10^{-6}, 4.8×10^{-6}, 6.0×10^{-6}, 7.2×10^{-6}, 8.4×10^{-6}, 9.6×10^{-6}, 10.8×10^{-6} and 12×10^{-6} mol·L^{-1}, respectively. BSA concentration applied was 1.04×10^{-6} mol·L^{-1}. Scan condition: Ex = 295 nm, Em = 305–580 nm; slits of both Ex and Em of compounds **1–4** were 5 nm. The blue line "a" was compounds **1–4**, which concentration was 12.0×10^{-6} mol·L^{-1} and had no BSA.

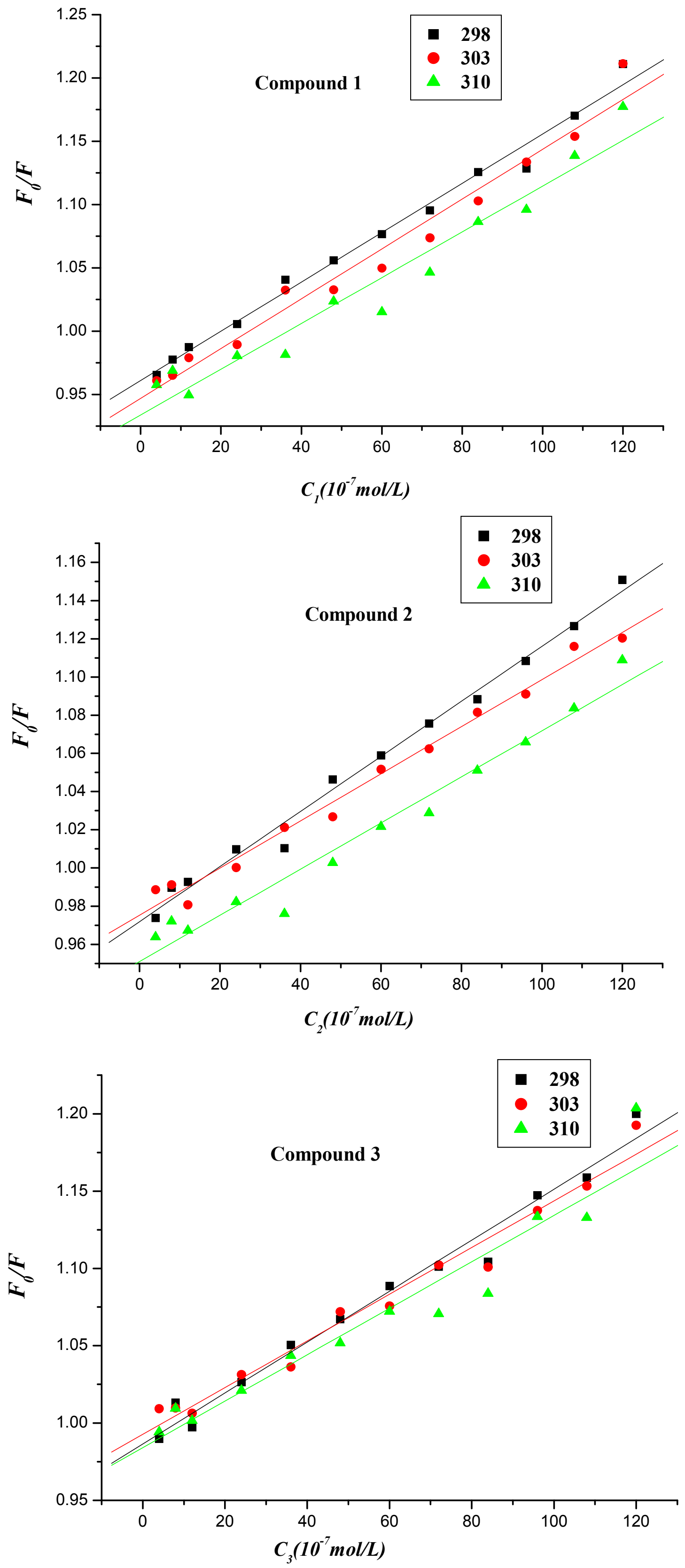

Figure S3. *Cont.*

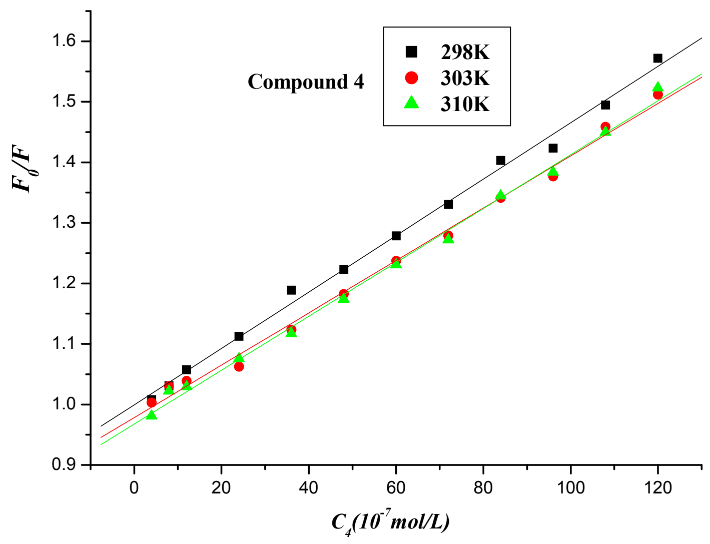

Figure S3. Stern-Volmer linear plot of fluorescence quenching of BSA by compounds **1–4** at different temperatures (Ex = 295 nm).

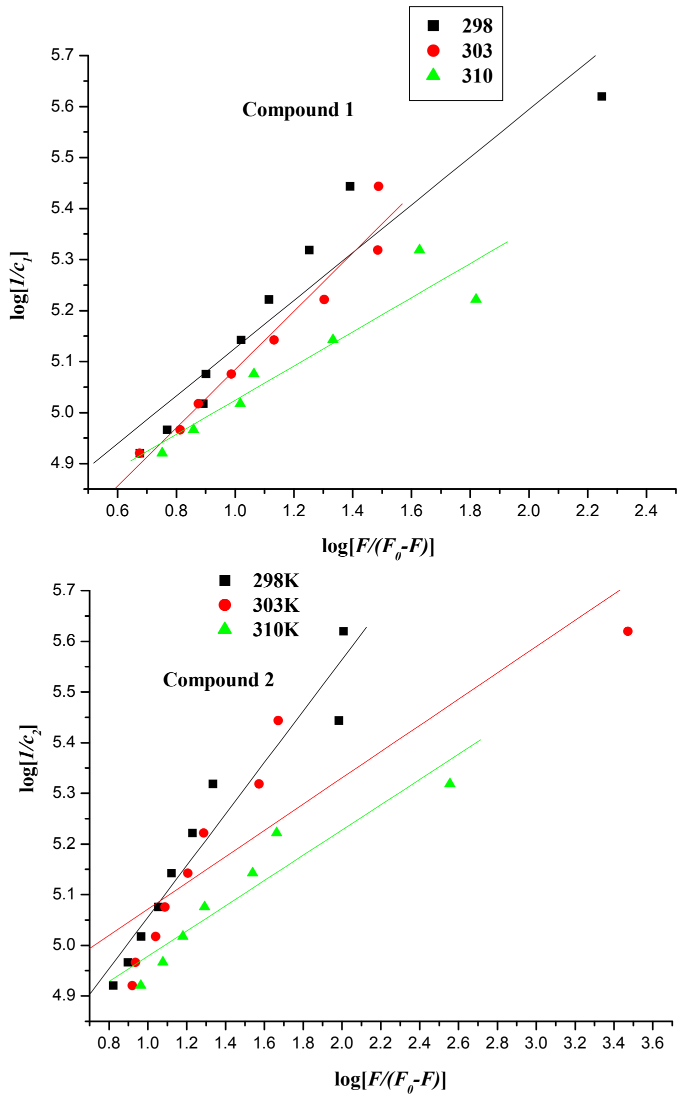

Figure S4. *Cont.*

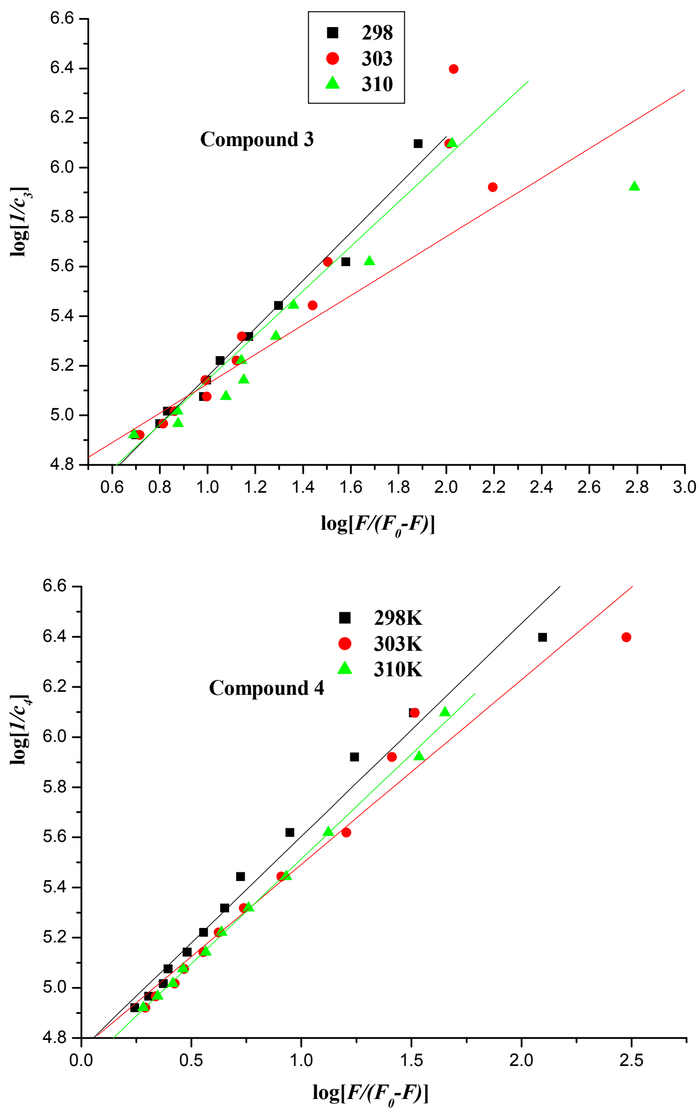

Figure S4. Linear plot of log [1/*c*comp. *vs.* log [*F*/(*F*0 − *F*)] of the interaction between compounds **1–4** and BSA at different temperatures (Ex = 295 nm).

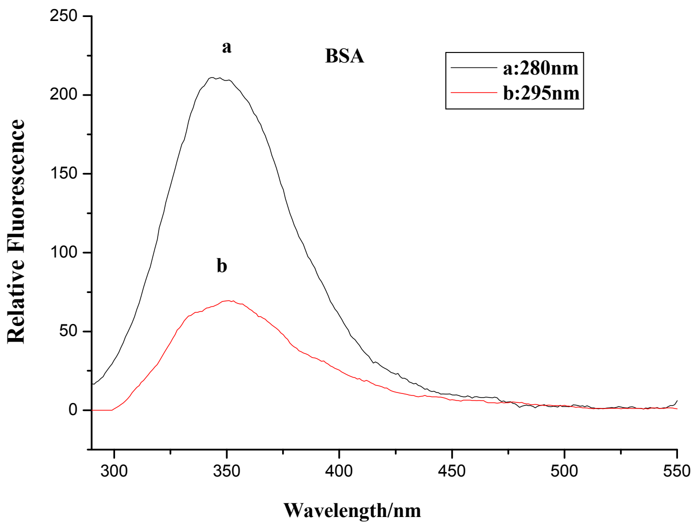

Figure S5. The Fluorescence of BSA at Ex = 280 and 295 nm, respectively; slits of both Ex and Em were 5 nm; *c* (BSA) = 1.04×10^{-6} mol·L^{-1}.

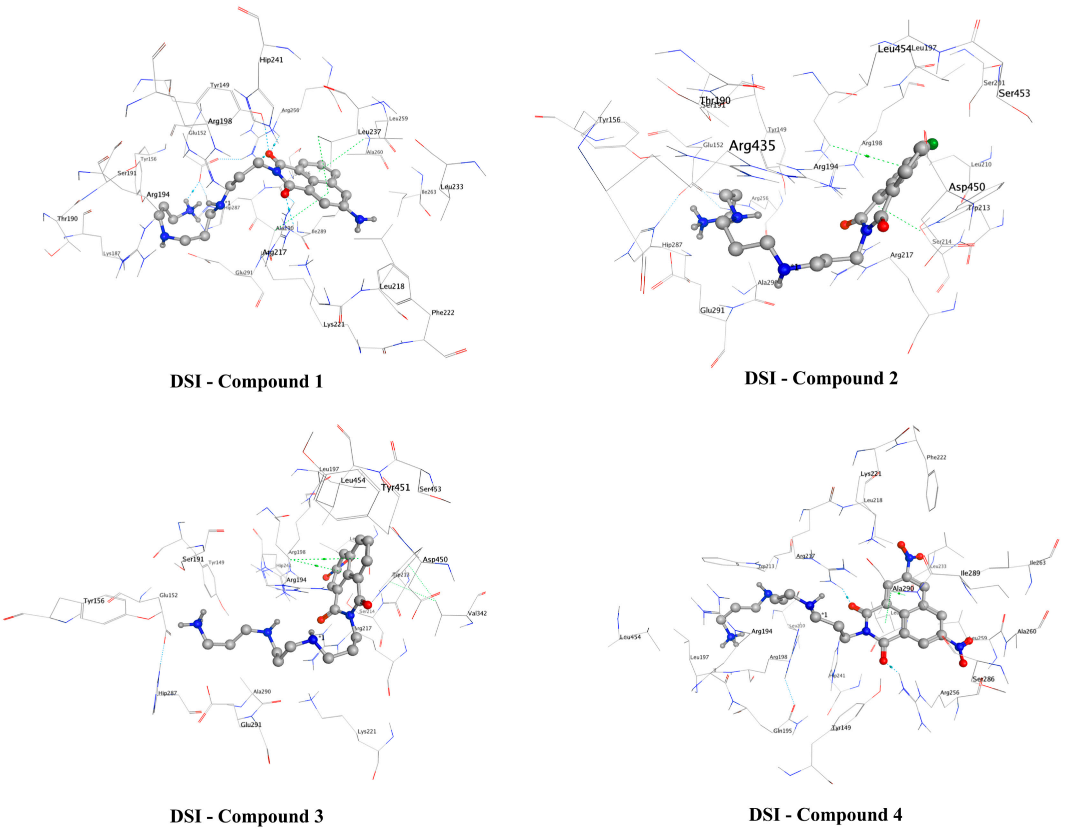

Figure S6. The 3D diagram of the best binding mode for the docking of DSI and compounds **1–4** according to the scoring.

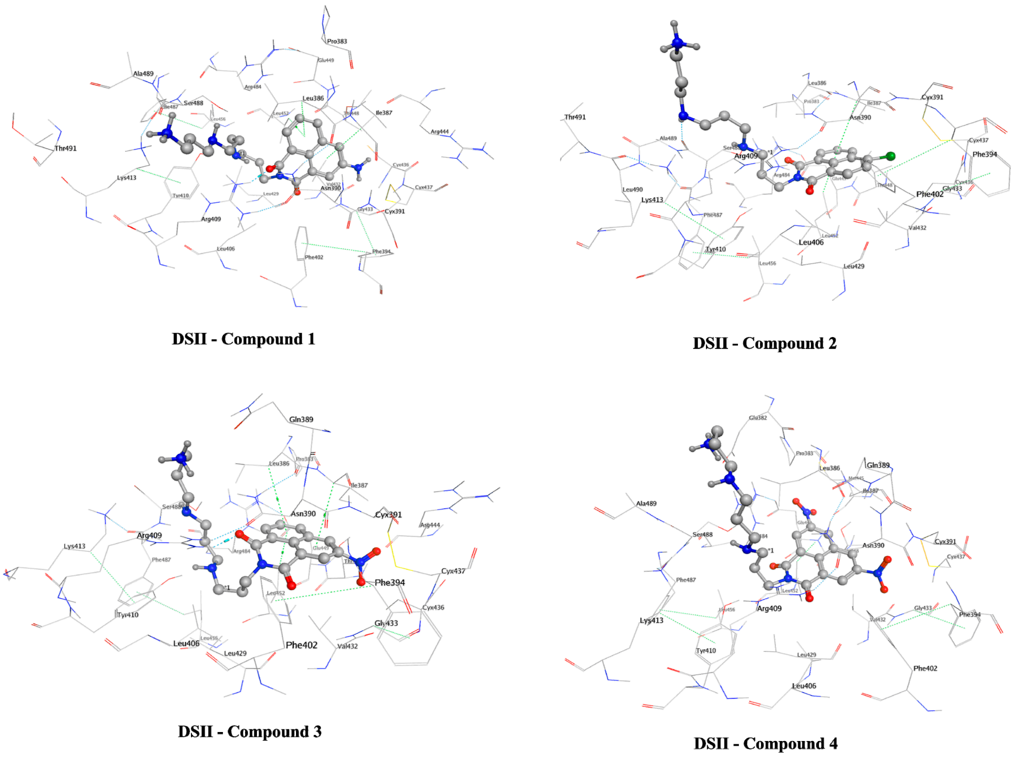

Figure S7. The 3D diagram of the best binding mode for the docking of DSII and compounds **1–4** according to the scoring.

Table S1. The fluorescence lifetimes of BSA and compounds **1**–**4**-BSA complex.

Medium	B_1	τ_1 (ns)	B_2	τ_2 (ns)	τ_0 (ns)	x^2 (%)
BSA	0.010	1.647	0.033	6.282	5.941	1.090
BSA + compound **1**	0.007	2.626	0.031	6.308	5.993	1.244
BSA + compound **2**	0.009	1.671	0.032	6.231	5.912	1.180
BSA + compound **3**	0.008	2.859	0.031	6.403	6.037	1.201
BSA + compound **4**	0.009	2.937	0.030	6.377	5.753	1.294

Note: *c* (BSA) = 1.04×10^{-6} mol·L^{-1}; *c* (compounds **1**–**4**) = 12.0×10^{-6} mol ·L^{-1}.

Table S2. Quenching constant of the interaction between compounds **1**–**4** and BSA at different temperatures (Ex = 295 nm).

Compound	*T* (K)	K_{sv} (L·mol^{-1})	K_q (L·mol^{-1})	*r*
	298	1.950×10^4		0.994
**1**	303	1.970×10^4	3.282×10^{12}	0.987
	310	1.810×10^4		0.975
	298	1.440×10^4		0.995
**2**	303	1.230×10^4	2.424×10^{12}	0.993
	310	1.210×10^4		0.984
	298	1.650×10^4		0.989
**3**	303	1.510×10^4	2.777×10^{12}	0.987
	310	1.500×10^4		0.963
	298	4.660×10^4		0.998
**4**	303	4.320×10^4	7.844×10^{12}	0.997
	310	4.450×10^4		0.998

Table S3. Binding constants and thermodynamic parameters of the interaction between compounds **1**–**4** and BSA at different temperatures (Ex = 295 nm).

Compound	*T*(K)	K_b (L·mol^{-1})	$\Delta G°$ (L·mol^{-1})	$\Delta H°$ (kJ·mol^{-1})	$\Delta S°$ (kJ·mol^{-1})	*r*
	298	4.556×10^4	−26.576	4.580	0.105	0.950
**1**	303	3.261×10^4	−26.179	4.580	0.105	0.977
	310	4.894×10^4	−27.831	4.580	0.105	0.938
	298	3.526×10^4	−25.941	26.781	0.177	0.964
**2**	303	6.488×10^4	−27.912	26.781	0.177	0.887
	310	5.358×10^4	−28.064	26.781	0.177	0.945
	298	1.554×10^4	−23.911	50.430	0.250	0.989
**3**	303	1.754×10^4	−24.618	50.430	0.250	0.957
	310	3.417×10^4	−26.905	50.430	0.250	0.917
	298	5.688×10^4	−27.126	−11.158	0.0536	0.990
**4**	303	5.709×10^4	−27.591	−11.158	0.0536	0.988
	310	4.778×10^4	−27.769	−11.158	0.0536	0.999
